# An integrated Pan-European perspective on coastal Lagoons management through a mosaic-DPSIR approach

**DOI:** 10.1038/srep19400

**Published:** 2016-01-18

**Authors:** Marina Dolbeth, Per Stålnacke, Fátima L. Alves, Lisa P. Sousa, Geoffrey D. Gooch, Valeriy Khokhlov, Yurii Tuchkovenko, Javier Lloret, Małgorzata Bielecka, Grzegorz Różyński, João A. Soares, Susan Baggett, Piotr Margonski, Boris V. Chubarenko, Ana I. Lillebø

**Affiliations:** 1 Biology Department & Centre for Environmental and Marine Studies (CESAM), University of Aveiro, Campus Universitário de Santiago, 3810-193 Aveiro Portugal; 2 CFE – Centre for Functional Ecology, Department of Life Sciences, University of Coimbra, PO Box 3046, 3001-401 Coimbra, Portugal; 3 NIBIO - Norwegian Institute of Bioeconomy Research, Ås, Norway; 4 Department of Environment and Planning & Centre for Environmental and Marine Studies (CESAM), University of Aveiro, Campus Universitário de Santiago, 3810-193 Aveiro Portugal; 5 DelPar Environment Consulting, Rotegatan 4, Linköping, 58752, Sweden; 6 Dundee Centre for Water Law, Policy and Science, University of Dundee, Dundee, Scotland, UK; 7 Department of Theoretical Meteorology and Weather Forecasts, Odessa State Environmental University, Odessa, Ukraine; 8 Department of Oceanology and Marine Nature Management, Odessa State Environmental University, Odessa, Ukraine; 9 Department of Ecology and Hydrology, University of Murcia, Murcia, Spain; 10 The Ecosystems Center, Marine Biological Laboratory, Woods Hole, MA 02543, USA; 11 Department of Wave Mechanics and Dynamics of Structures. Institute of Hydro-Engineering Polish Academy of Sciences, Gdansk, Poland; 12 Department of Coastal Engineering and Dynamics, Institute of Hydro-Engineering Polish Academy of Sciences, Gdansk, Poland; 13 National Marine Fisheries Research Institute, Gdynia, Poland; 14 Atlantic Branch of P.P.Shirshov Institute of Oceanology of Russian Academy of Sciences, Kaliningrad, Russia

## Abstract

A decision support framework for the management of lagoon ecosystems was tested using four European Lagoons: Ria de Aveiro (Portugal), Mar Menor (Spain), Tyligulskyi Liman (Ukraine) and Vistula Lagoon (Poland/Russia). Our aim was to formulate integrated management recommendations for European lagoons. To achieve this we followed a DPSIR (*Drivers-Pressures-State Change-Impacts-Responses*) approach, with focus on integrating aspects of human wellbeing, welfare and ecosystem sustainability. The most important *drivers* in each lagoon were identified, based on information gathered from the lagoons’ stakeholders, complemented by scientific knowledge on each lagoon as seen from a land-sea perspective. The DPSIR cycles for each *driver* were combined into a mosaic-DPSIR conceptual model to examine the interdependency between the multiple and interacting uses of the lagoon. This framework emphasizes the common links, but also the specificities of *responses* to *drivers* and the ecosystem services provided. The information collected was used to formulate recommendations for the sustainable management of lagoons within a Pan-European context. Several common management recommendations were proposed, but specificities were also identified. The study synthesizes the present conditions for the management of lagoons, thus analysing and examining the activities that might be developed in different scenarios, scenarios which facilitate ecosystem protection without compromising future generations.

Coastal and transitional regions have long been attractive for human populations due to their high availability of ecosystem services and multiplicity of uses; this has placed these systems under severe anthropogenic stress^1^,^2^,^3^,^4^. The special challenges facing coastal regions, has, during recent decades, highlighted the need of policies for marine protection that follow an Ecosystem Approach^5^,^6^,^7^. An Ecosystem Approach is here taken to mean that the management of human activities should ensure the ecological and environmental health of the ecosystems and a sustainable use of their services, while maintaining ecosystem integrity^8^,^9^,^10^. However, an efficient and effective management of marine ecosystem also needs to ensure that the different intervening users of the system are heard^4^,^11^,^12^,^13^ and also that it addresses the interacting uses of the ecosystem^7^.

The aim of the present work is to assess contemporary conditions regarding drivers, their impacts and potential management options on European lagoons. The selected European lagoons (Ria de Aveiro, Mar Menor, Tyligulskyi Liman and Vistula) have different characteristics regarding hydrology, land use, governance issues, among others, covering the main European geographical locations (respectively, Atlantic ocean and the Mediterranean, Black and Baltic seas). The aim is to identify current problems and concerns, policy needs, and to propose recommendations for management that specifically address the experience and views of the local users of the lagoons. The preliminary collection of information took into account the present knowledge base using existing data on environmental conditions, it identified knowledge gaps, protection policies and development needs, and examined how these were addressed by the different actors using the coastal lagoon or contributing for its management: from research centres, public and private institutions to the lagoons’ end-users. This constitutes one of the major novelties of the work presented here and demonstrates that it is possible to enhance connectivity between research and environmental management in a lagoon’s context using a proactive approach to water issues, an approach which also ensures a more efficient use of existing research results. This active involvement of stakeholders and the inclusion of the public concerns in water management issues have been encouraged by several authors^11^,^14^,^15^, as the public support is considered essential to implement changes.

The ultimate goal of this paper is to structure information regarding major drivers and their impacts to support the description of management recommendations for lagoons’ management into a Pan-European context, using the four selected case study lagoons. For this purpose, we followed a DPSIR approach (*Drivers-Pressures-State change-Impacts-Responses*), underpinning the Ecosystem Approach, by integrating human wellbeing, welfare and ecosystem sustainability aspects (society, economy and ecology)^7^,^16^. From this perspective, DPSIR becomes an essential tool to structure and identify the causal relationships between *drivers* of change and their *impacts,* and through this process, to identify guidelines for environmental management. The aim is to promote resistance and resilience to environmental change in a way that engages natural, social and economic sciences^16^,^17^,^18^. When applied in a participatory and multi-methodology framework such as the one presented here, the process also considers conflicting stakeholders claims and facilitates an explanation of how different management options might impact ecosystem services and stakeholders in general (e.g.^10^,^15^). This approach ensures that lagoons are perceived as essential natural resources on which humans depend in a variety of forms, enables end-users vision to be included in the decision making processes, and in this way foster agreements regarding management options

## Material And Methods

### Study sites

**Figure 1:**
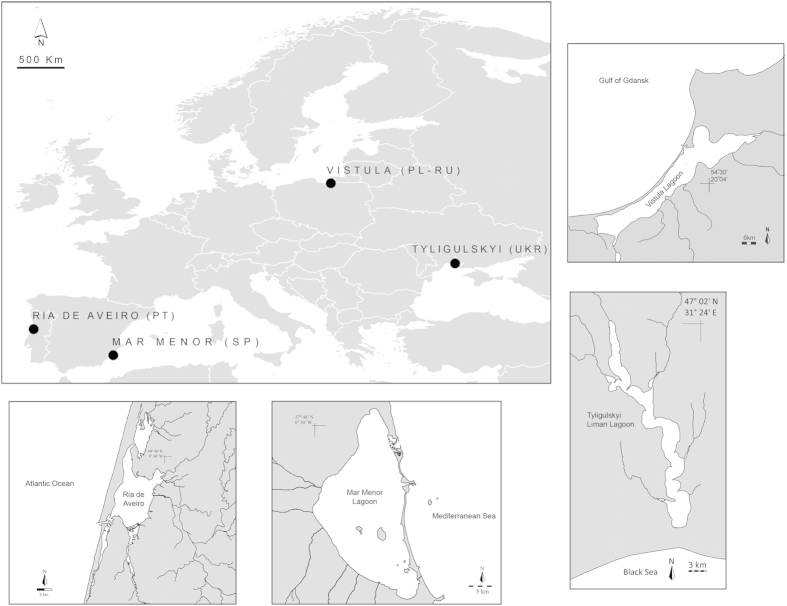
The geographic distribution of the LAGOONS case studies in the European continent and maps of each lagoon. Maps were individually generated with ARC-Gis 9.2 software and compiled CorelDRAW 12 software.

Four lagoon study sites were selected (Fig. 1), located in distinct European geographical locations, connected to four different seas and with different characteristics (Table 1): 1) Ria de Aveiro Lagoon in the Atlantic Ocean (Portugal), a mesotidal southern lagoon with an area of 83 km^2^, with large intertidal flat areas exposed during low tide and a large salinity range. The lagoon is characterized by a temperate maritime climate; 2) the Mar Menor Lagoon in the Mediterranean Sea (Spain), a medium-size Mediterranean hypersaline lagoon, with an area of 135 km^19^, in a semi-arid region, characterized by warm and dry weather conditions; 3) the Tyligulskyi Liman Lagoon in the Black Sea (Ukraine), with an area of 129 km^2^, connected to the sea by an artificial channel which is operational only 3-4 months per year. The lagoon is characterized by a temperate and continental climate regime and usually a low salinity range, that has been increasing during the last decades; and 4) the Vistula Lagoon in the Baltic Sea (Poland/Russia), the second largest lagoon the Baltic Sea with an area of 838 km^2^, and low salinity. The Vistula Lagoon is under the influence of both maritime and continental climate conditions, depending on the region, and can attain high annual air temperature amplitudes (from −31 °C to 36 °C). It is also characterised by a transboundary management regime shared by Poland and Russia. All four lagoons are connected to the sea by a single narrow and shallow entrance, artificially controlled for Tyligulskyi Liman Lagoon, and with different water fluxes, due to natural river flow and water management issues^20^. The lagoons share some common *drivers*, albeit with different levels of social and economic importance and ecological *impact* in each lagoon. All lagoons play important ecological roles and have been recognised in numerous national and international protection tools/instruments, such as Ramsar sites (e.g. Mar Menor and Tyligulskyi Liman), Nature 2000 Network (Ria de Aveiro, Mar Menor and the Polish part of the Vistula Lagoon), which also integrates ‘Specially Protected Areas’ – SPA, ‘Sites of Community Importance’ – SCI and the Birds Directive 79/409/CEE. For more details on each Lagoon case study area, please see^21^ and the deliverables available in http://lagoons.web.ua.pt.

## Methods

For the development of the integrated management recommendations we used the DPSIR framework (*Drivers-Pressures-State change-Impacts-Responses*^7^). *Drivers* (D) are anthropogenic activities that may generate environmental effects (considering changes in social, economic or cultural aspects). *Pressures* (P) refer to a direct and quantifiable effect of an anthropogenic *driver* in the system, where causes of potential adverse effects come from within a system and require local, regional, and/or international management. The *State change* (S) is the environmental condition of the lagoon (i.e. physical, chemical and biological characteristics) resulting from both natural and anthropogenic pressures. *Impact* (I) is defined as the impact caused by the changes of the *State* on human wellbeing, welfare and sustainability, therefore combining social, economic and ecological aspects. More specifically, it considers the impacts on society, in association with ecosystem services^7^, i.e. provisioning, regulation and maintenance, and cultural services as defined by^22^. *Responses* (R) are interventions to minimise or mitigate negative effects of an impact and should meet the ten tenets for environmental change: “Environmentally/ecologically sustainable; Technologically feasible; Economically viable; Socially desirable/tolerable; Legally permissible; Administratively achievable, Politically expedient, Ecologically Sustainable, Ethically defensible (morally correct), Culturally inclusive and Effectively communicable”^23^. Finally, we have considered *natural change* as potentially impacting the *state* in a DPSIR cycle. Natural change is defined as *pressure* originated from a natural source, for which local management systems cannot address the causes of change, but can only address their consequences (e.g. climate change^6^,^7^).

For the application of the DPSIR we combined knowledge from different scientific disciplines with information obtained through participative approaches involving local populations, aiming to integrate environmental and social-economic aspects in our assessment. The participatory approaches with the local population were conducted simultaneously in the four case study lagoons, following a chronologic sequence of different methodologies with specific goals, i.e. focus groups and citizens’ juries. Focus groups and citizens’ juries meetings were held for each case study lagoon to ensure that a broad set of relevant local stakeholders’ (decision makers, managers, end-users and general public) groups were engaged (e.g. for Ria de Aveiro^24^; for the Vistula Lagoon area^25^). Details on the focus groups and citizen juries selection and procedure can be found in^24^,^26^. In these meetings, some initial open questions were structured to guide the discussion into the uses of the lagoon, their most important aspects, main changes, development in and around and management issues. Insights were identified into what the lagoons’ stakeholders viewed as the main features of the lagoon, concerns regarding any issues or problems, the location of these in the lagoon and what future changes, if any, they were anticipating or would like to take place^27^. All participants signed a written informed consent, authorizing us to use the results from the Focus Groups and Citizen Juries for each of the case study lagoons. The consents were written in the case study lagoon local language following the usual procedure for Focus Groups and Citizen Juries participatory processes for the four lagoons. These data were analysed anonymously. The outputs of these meetings provided an ongoing platform for local participation, knowledge building and achieving the input sought through the active engagement of the stakeholders within each of the four case study areas. In this way, we ensured that the DPSIR, and in particularly the recognition of the main *drivers,* their *pressures,* consequent *state, impacts* and potential *responses* represented the local society’s vision for the lagoon. This way, we ensure to include knowledge through local experience and observation, regarding the environment and social-economic aspects.

**Figure 2:**
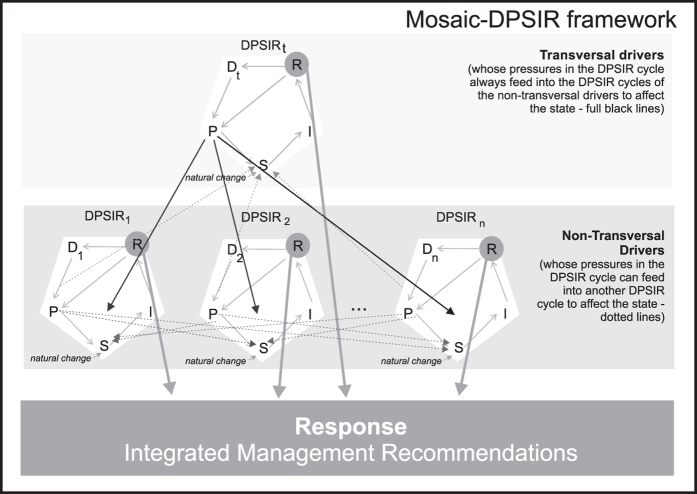
Conceptual scheme for the mosaic-DPSIR for an ecosystem (adapted from^7^), distinguishing between transversal *drivers*, as the ones whose DPSIR cycles feed on all other DPSIR cycles, and the non-transversal *drivers*, as the ones whose DPSIR might feed on the others. *Responses* from all DPSIR cycles should be integrated taking into account the interactions among DPSIR cycles.

For the *drivers* identified by the stakeholders as having higher importance in the lagoon’s ecological and social-economic activities, DPSIR cycles were produced for each single *driver,* where all DPSIR elements were scrutinized, taking into account the different sources of information. The participatory methods were complemented by the existing knowledge on the lagoons’ physical, chemical, biological characteristics and management system (references for the existing scientific knowledge included in the supplementary information). These DPSIR cycles were then subsequently combined to produce a mosaic-DPSIR, adapting the concept of mosaic in ecology, which consists of numerous small pieces (or elements) fitted together. In the mosaic-DPSIR elements of the DPSIR cycles could have multiple interactions among each other: *pressures* of one DPSIR cycle may feed on the *pressures* and *state* from another DPSIR cycle, and, as such, *responses* need to be organized in an integrated management framework. We have also distinguished between the DPSIR cycle that always feed on the *pressures* of all DPSIR identified cycles – hereafter named as transversal drivers, from the ones that might affect other DPSIR cycles – non-transversal ones (Fig. 2).

All DPSIR cycles and mosaic-DPSIR’s for each lagoon are available as supplementary information. The mosaic-DPSIR for each lagoon will be integrated to propose recommendations within a Pan-European context, specifying the differences and the common links among *responses.*

## Results And Discussion

### Overview of the major ***drivers***

**Figure 3:**
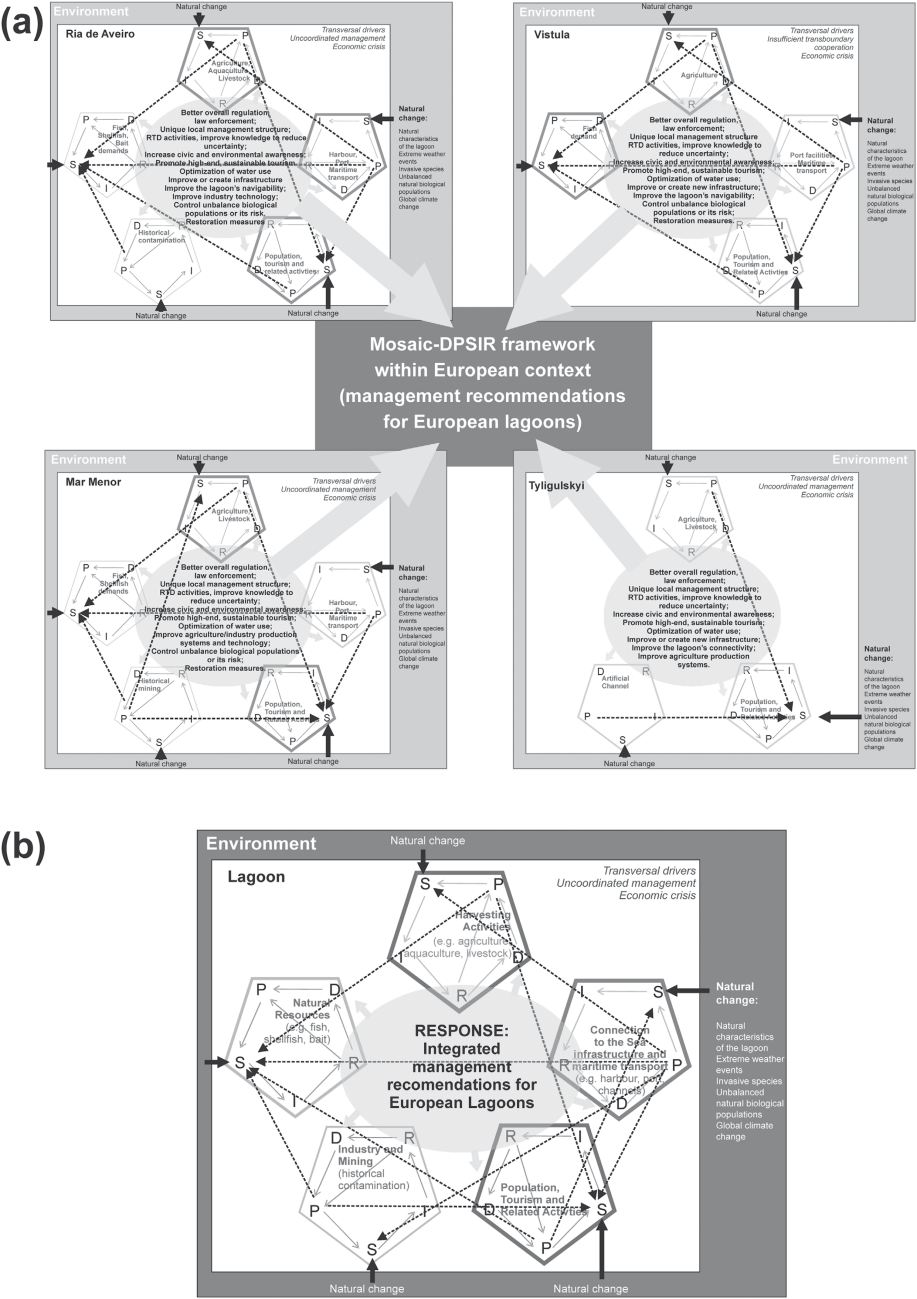
Mosaic-DPSIR cycles for the LAGOONS case studies, showing the multiple interactions among DPSIR cycles (dotted arrows) and the influence of natural change on the *state* (black arrows), and that *responses* from each cycle should be combined into a common integrated *responses* –(**A**) for each lagoon, with emphasis on the *drivers* with higher social-economical expression in the lagoon; (**B**) integrated for Pan European context, using information from the four lagoons and with emphasis on *drivers* appearing in all lagoons. For the integrated management recommendations please see Fig. 4.

For all four case studies there are several *drivers* common to all the lagoons, although the resulting *pressures* changed for each lagoon, depending on the socio-economic development in the region, which resemble to *drivers* and *pressures* in other coastal lagoons worldwide^28^. At least two *drivers* were identified in all lagoons as having clear multiple interactions and affecting the *state* in all DPSIR cycles, and are therefore considered by us as transversal *drivers* (Fig. 2). These are uncoordinated management for all lagoons and insufficient transboundary cooperation in the case of the Vistula Lagoon, and the economic crisis for all lagoons (Fig. 3, Fig. S2, S4, S6, S8 of supplementary information). Management recommendations should necessarily take into account this interaction (mosaic-DPSIR framework, Fig. 2).

Regarding the other *drivers, pressures* generated in one DPSIR cycle can feed into another DPSIR cycle, where it may interact with the *pressure* from that cycle to influence the *state.* However, the DPSIR of these *drivers* do not necessarily affect all other DPSIR cycles – non-transversal *drivers* in the mosaic-DPSIR (Fig. 2). Potentially impacting all lagoons, directly with either positive or negative *impacts* are population and demographic growth, tourism and related activities, and the lagoon-sea infrastructure connection and maritime navigation (harbours, port, artificial channels, Table 2, Fig. 2A). Agriculture is also a *driver* identified in the four lagoons, but has higher economical importance for Mar Menor and Ria de Aveiro (Table 2, Fig. 2A). Within harvesting activities, aquaculture is an important activity, especially for Ria de Aveiro. Fish, shellfish and bait catches are the main demands on natural resources for some of the lagoons (Table 2). In the Ria de Aveiro, aquaculture and fishing are extremely important activities, both economically and societally and as part of the region’s cultural identity, but these are less important in the other lagoons. Industry is not extensively developed in some lagoons (e.g. Tyligulskyi Liman and Vistula Lagoon), and for the other lagoons *impacts* correspond to historical contamination patterns in restricted areas (e.g. industry in Ria de Aveiro, mining in Mar Menor, Table 2).

All *drivers,* transversal and non-transversal ones, will be discussed in more detail below, together with the *natural changes*, which have been identified in all lagoons (Table 3) and potentially affect the *state change* from all identified *drivers* (Fig. 3).

## Transversal drivers

### Uncoordinated management and insufficient transboundary cooperation

**Figure 4:**
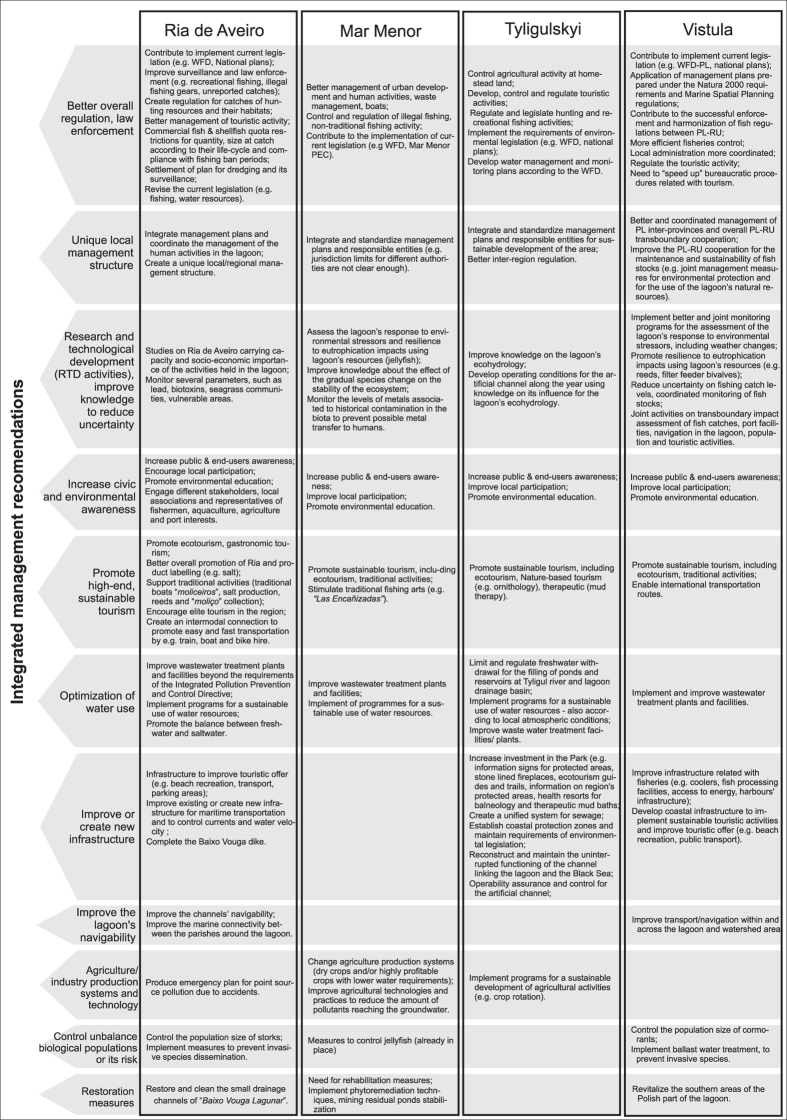
Integrated management recommendations for each lagoon for their present condition, highlighting common general responses and specific responses for each lagoon, taking into account information obtained from the lagoons’ stakeholders, complemented by scientific knowledge on the lagoons’ physio-geographical and ecological characteristics.

During the participatory process the stakeholders identified ‘Uncoordinated Management’ for all lagoons and ‘Insufficient Transboundary Cooperation’ for the Vistula as problematic issues. As mentioned above, we considered these as being transversal *drivers* as they have clear multiple interactions with all the other *drivers*. For all lagoons, there are several institutions responsible for the management for the different sectors and administrative regions; in the case of the Vistula Lagoon this is especially notable as here there are two different countries. When the lagoon is shared between EU and non-EU countries the need for coordination becomes especially important^25^,^29^. The need for a unified management coordination framework or a harmonized cooperation between transboundary lagoons was identified by the stakeholders (Fig. 4), a concern that has been raised by others^30^,^31^. However, the way to achieve this goal is far from consensual. Some of the reasons pointed for the lack of cooperation between the different authorities engaged in managing the lagoons were related to their mandates time frame and possible inter-organisational rivalries^32^. The Vistula Lagoon case study showed that even the existing transboundary commission organised under an intergovernmental agreement is not able to cover all issues without the involvement of a wide spectra of stakeholders and public. Stakeholders participatory process clearly highlighted that there are major challenges to improving the cooperation between the different authorities engaged in managing the lagoons, and between these and the local population^32^.

### Economic crisis

The economic crisis of the last years in Europe was also treated as a transversal *driver,* as it was expected to change some of the existing *pressures* and consequently also change the *state* and *impact* of all *drivers*, due to the lower economic resources and lack of investments to implement needed changes. Unemployment, for example, may increase illegal activities such as poaching; lack of law enforcement, surveillance and control may lead to the use of illegal fishing equipment and a lack of human resources to control illegal fishing. In the Ria de Aveiro (Fig. 1SD, 3SD) unreported fish and shellfish catches sold directly to restaurants were identified by the stakeholders as a major problem (Fig. 1.4s). Limited economic resources may also lead to lower investments in wastewater treatment facilities, reduce the operationalization of programs for the sustainable use of water use resources, decrease agricultural development and sustainable tourism development, and lead to a lack of implementation of monitoring programmes (e.g.^33^). These can be seen in the management recommendations (Fig. 4). These aspects, together with projections of socio-economic development for each country, need to be taken into consideration when proposing recommendations for the management of the lagoons.

### Non-transversal drivers

#### Population, Tourism and Related Activities

Population density and growth were identified as important *drivers* in the four-case lagoons. This *driver* has also been identified in other coastal lagoons worldwide^2^,^34^ and can be seen as a result of a high level of human population concentrations near coastal regions^30^,^35^. This is due to the high availability of ecosystem services in these regions. The population concentration in coastal regions increases the likelihood of *pressures* related to the intensity of urbanisation, water use and wastewater management; the latter with different degrees of treatment (Fig. S1A, S3A, S5A, S7A from supplementary information). In the two southern-most located lagoons, Ria de Aveiro and Mar Menor, the enhancement of wastewater treatment plants and the implementation of legal frameworks for water use within the context of European water policies in the last decades has contributed for the improvement of ecological conditions in the lagoons. In the two northern lagoons, Tyligulskyi Liman and Vistula, there are still several problems related with waste disposal and insufficient wastewater treatment (Fig. S5A, S7A).

Population was highly associated with tourism (Fig. S1A, S3A, S5A, S7A), especially in the Mar Menor, where the population can increase up to 10-fold during the summer season (Fig. S3A). In the other lagoons, tourism activities are also seasonal though to a lesser degree than Mar Menor. The *pressures* and *impacts* on the *state* in the lagoons resulting from the different *drivers* depend on the country’s policies, level of development, and on the natural characteristics of the lagoon (natural change, Table 3). However, this can result in eutrophication, degradation of the ecological condition and ‘landscape’ changes of the lagoon, also in combination with other *drivers*’ impact (e.g. agriculture in the mosaic-DPSIR, Fig. 3). In the Tyligulskyi Liman Lagoon, eutrophication is site-specific and seasonal, resulting in phytoplankton and macrophyte blooms during summer (Fig. S5A, S7A). In Mar Menor and in the Vistula Lagoon, eutrophication is more generalized, resulting in phanerogam regression and progressive substitution by benthic perennial macroalgae in Mar Menor^36^ (Fig. S3A, B), or affecting the whole ecosystem functioning in the Vistula Lagoon (Refs 37 and 38, Fig. S3A, B). Most of the Ria de Aveiro has been judged as having a moderate degree of eutrophication and low human interference^39^. As for other common *impacts*, the lack of regulation of tourism and recreational activities often results in the occurrence of illegal activities such as illegal fishing and hunting in all lagoons, and non-regulated construction, Fig. S1A, S3A, S5A, S7A. This can of course cause conflicts between and within the economical sectors and *drivers* – in the mosaic-DPSIR. For example, incompatibilities between professional and recreational fishing due to the unbalanced number of licences, negatively affect fish, shellfish and bait resources (i.e., antagonistic interactions). These concerns were expressed by the stakeholders of all lagoons through the participatory meetings, and the need for regulation and protection tools was stressed^24^ and check also *responses* in Fig. S1A, S5A, S7A). However, the stakeholders also emphasised positive aspects related to tourism, mostly associated to increased employment and wealth creation. In fact, for all lagoons, sustainable tourism activities were considered as having the potential to grow, as long as they were adequately delimited and controlled (*Responses,* S1A, S5A, S7A, Fig. 4). Lagoon ecosystems are generally characterized by high biodiversity, high aesthetic value regarding the landscape, cultural identity, including several traditional activities (e.g., salt production in Ria de Aveiro, angling in the Vistula Lagoon), which are clear signs of the potential interest for Nature-based tourism (e.g. birds watching, excursions) and other forms of touristic activities (e.g. sports, mud therapy, gastronomy). However, an investment in sustainable tourism would also need an investment in raising stakeholders awareness (e.g. environmental education programmes) and, in some cases, development of infrastructure such as trails for excursions and identification of the protected areas, in order to ensure that the lagoons’ natural resources are respected and preserved (*Responses*, Fig. 4).

#### Harvesting Activities

The most important harvesting activities involving human mediated production with the potential to impact the lagoons are agriculture, especially for Mar Menor, and, to lower economic extent, livestock and aquaculture. The *impacts* from these harvesting activities are possible water quality degradation, eutrophication, habitat and biodiversity loss, among others (Fig. S1B, S3B, S5B, S7B). However, since agriculture is a highly important economic activity, its management necessarily needs to compromise between the economy, biological conservation and the maintenance of a good ecological status (e.g. WFD). Examples of possible *responses* include improving agriculture practices and implementing programs to develop sustainable agricultural activities, taking into account local climate conditions, which will prevent water pollution and excessive water needs (e.g. Fig. 4). This *driver* is a clear example of the ways that the mosaic-DPSIR can be used and integrated with an Ecosystem Approach, as the harvesting activities affect the *state* of other *drivers*, such human population and tourism and the natural resources demand. Agriculture’s nutrient discharge may also intensify eutrophication problems; and incompatibilities between salt production and aquaculture, which involve sharing the same water and may also lead to conflicts. However, aquaculture could also contribute to a solution to the over-exploitation of fish and shellfish resources (Fig. 4). Evidently, the implications of this *driver* for the *state* of other *drivers* clearly showcase the need for an integrated management plan across all *drivers*.

#### Connection to the Sea

For all four lagoons, the connection to the sea is extremely important as this determines water exchange, hydrodynamics and physico-chemical conditions, including salinity and nutrient content in the lagoon^40^. Thus, the connection to the sea influences the lagoon water quality and resources such as flora and fauna composition and abundance. These in turn need to be included in the management recommendations as identified by the stakeholders. It is necessary to understand and take into account the impact of fauna compositional changes for the stability and economy of the lagoon (e.g. fish and benthic assemblages, including invasive species appearance), as well as to include aspects of seagrass decline and the need for the stabilization of water level (Fig. S1C, S3C, S5C, S7C). For Ria de Aveiro, Mar Menor and Vistula, harbour and navigation channels are maintained through dredging, which results in several *impacts* (Fig. S1C, S3C, S7C), derived from the changes in the sediment dynamics, balance between erosion and accretion processes and water regime^41^. However, in the Ria de Aveiro, the commercial harbour and the existence of maritime navigation are intensive and important commercial activities, considered extremely important for the regional and national economy in Portugal. The *pressures* from the ‘Connection to the Sea’ DPSIR, however, feed into another DPSIR cycles, influencing the *state* of those cycles (mosaic-DPSIR), resulting in conflicts between the harbour’s interests and the interests of other activities such as fishing, water-based tourism and recreation, and agriculture. In the case of agricultural land, salinisation is a major problem. This highlights the need to engage the Ria’s different stakeholders in order to determine and formulate a common action plan, a plan that acknowledges their needs and the needs of the environment itself (Fig. 4). For the Tyligulskyi Liman Lagoon, the connection to the sea is maintained by an artificial channel that is operational during 3-4 month per year until sanding up from the sea-side^42^. For both the Vistula and Tyligulskyi Liman lagoons, water exchange is highly variable and dependent on water level variations in the adjacent marine area and on local weather (wind velocity and direction, precipitation, surface evaporation, ice cover periods). It is also dependent on the morphology and hydrology of the lagoons, i.e., *natural change*. For these lagoons, it is crucial to understand the lagoon’s ecohydrology, to improve the lagoon’s connectivity in order to enhance the exchange of water between lagoon-sea for Tyligulskyi Liman and to refine the navigability of Vistula Lagoon when needed. Improving the lagoon’s navigability via dredging and/or the widening of the inlets that connect the lagoon with the adjacent ocean might however have unexpected and, in some cases, undesirable consequences for the lagoon’s ecological processes and functioning. An example of these impacts can be observed in the Mar Menor. In the early 1970s one of the lagoon inlets (*El Estacio*) was dredged and widened to make it navigable. As a consequence, temperature and salinity ranges became less extreme, allowing the colonization of the lagoon by several Mediterranean species, including the macroalgae *Caulerpa prolifera*, or the undesirable jellyfish species *Cotylorhiza tuberculata* and *Rhizostoma pulmo*^43^,^44^. These colonisations caused a profound impact on both water and sediment quality in the lagoon and fauna communities (e.g.^44^,^45^,^46^), with negative consequences for local tourism.

#### Natural Resources (fish, shellfish and bait)

Most of the economically viable natural resources in the lagoons are connected with commercial fishing and shellfish and bait collection, especially in the Ria de Aveiro and Mar Menor (Table 2). The major *pressures* resulting from fishing are related to the increased harvesting of the target populations of fish, shellfish and bait. This leads to their decline, a decline which may also be augmented by illegal fishing and the use of illegal equipment, lack of regulation and insufficient surveillance (Fig. S1D, S3D, S7D). The decaying of traditional fishing has also *impacts* as contributing for the loss of cultural identity of the region. In a similar way to the previous *drivers*, fishing has high local economic and social importance and is perceived as additional income for many families. The social aspects of fishing culture, including traditional fishing and recreational fishing, has the potential to be developed as a sustainable local activity, as long as it is sufficiently regulated. Nowadays, however, there are several conflicts between professional and recreational fishing and increased efforts are needed to raise environmental awareness, including understanding of the need to enforce periods when fishing is not allowed, limits on the size of retained fish, and stricter prevention of unreported catches (Fig. 4).

#### Industry and Mining Activities

As noted above, today, industrial activities are relatively controlled in Ria de Aveiro and Mar Menor and are not extensively developed in Tyligulskyi Liman and Vistula Lagoons. However, there is historical contamination or <10% of point source pollution in restricted areas in Ria and Mar Menor (Fig. 1SE, 3SE,^39^,^46^). In addition, industrial activities are now regulated by restrictive rules in order to control environmental contamination. Despite this, and without going into discussion of the effectiveness of the implementation of these regulations, there are still potential risks of site-specific water and food chain contamination These increase after extreme weather events such as storm surges and floods (natural change *pressures*), or man-induced activities such as dredging and accidental spilling. An accidental food chain contamination would restrict other *drivers*, such as catches of fish, shellfish and bait (Fig. 3). *Responses* therefore need to address these risks. The *impacts* of such *pressures* highlights the need for emergency plans to manage accidental pollution. These plans need to guarantee the implementation of national and international legislation aimed at preventing contamination, providing for rehabilitation measures and the monitoring of potential contamination in the system (e.g. Mar Menor, Fig. 3SE), and through the food chain (Fig. 4).

### Natural change

Finally, *natural change* (Table 2) also affect the *state* from the anthropogenic *drivers* and cannot be dissociated from the mosaic-DPSIR (Figs 2,3). The *natural change* generally fall into the main categories (Table 2) of 1) natural variability, including geo-, morpho- and hydrological characteristics of the lagoon, with special relevance for the ecohydrology issues and coastal erosion; 2) ecological issues, including unbalanced natural populations or the invasive species occurrence as a result from an extension of their natural geographical range due to climate changes; 3) climate, including extreme weather events (e.g. floods, droughts, storm surge events, prolonged ice-cover periods) and future climate change, to which coastal lagoons may be particularly vulnerable^3^,^40^,^47^. These categories are themselves interconnected, for instance, ecohydrology and coastal erosion are expected to change with the climate, and climate might also “create” opportunities for invasive species proliferation or lead to unbalanced natural populations^48^. The main feature of the *natural change* is that only their consequences can be managed^6^,^7^. In the case of the natural characteristics of the lagoon we can anticipate potential *responses* for those consequences-e.g. managing vulnerable infrastructure on the boundary between lagoon/sea according to the lagoon ecohydrology and weather interactions (Fig. 4). With regard to future climate change projections (e.g. global warming, sea-level rise, altered precipitation patterns^49^), however, their precise effects are unknown, particularly with regard to the provision of ecosystem services^48^. Nevertheless, this study provides us with present knowledge, taking into account the values, norms and perceptions from a variety the lagoons’ users that allow us to recognize priority development areas and, thus, to create a decision support framework for the integrated management of lagoons that can be used in a climate change context.

### Mosaic-DPSIR and the Pan-European view

In the previous section, we have shown examples where the *pressures* generated in one DPSIR cycle can feed into another DPSIR cycle, where it would interact with the *pressures* from that cycle to influence *state* (Fig. 3), which in turn might generate conflicts. *Responses* for the management of the whole lagoon necessarily need to address this causality and these feedback loops between different *drivers* and respective DPSIR, which is the basis of the mosaic-DPSIR approach (adapted from^7^). To accomplish this, we present a series of recommendations for each lagoon that result from the stakeholders’ participatory process complemented by present lagoons’ knowledge and research, and take into account all *drivers*, transversal and non-transversal ones and also the *natural change* (Fig. 3 for common *responses* in each lagoon, Fig. 4 for specificities).

Moving to a Pan-European context, we have seen that there might be feedback loops within *pressures* and *state* from all *drivers* (Fig. 3B). So, what are the management needs for coastal lagoons within a Pan-European context? Essentially the proposed mosaic-DPSIR for each lagoon enables us to understand in a simplified manner which *drivers* have the potential to increase in the future without severely compromising the environment and the economy, and how this growth should be managed, taking into account the interconnections between different *drivers, natural change*, and consequent *impacts.* Several recommendations were proposed by the stakeholders from our four case lagoons which can be broadly summarised as better governance, improved awareness, engagement and commitment of the lagoons’ stakeholders to the lagoons issues, and improved knowledge with regard to the lagoon’s hydrological and ecological characteristics (Fig. 4), i.e., all issues that have been raised before, by several other authors (e.g.^4^,^7^,^13^,^34^). However, more important than the general recommendations are the specificities of the measures proposed, which have deliberately been designed taking into account ecological and socio-economic aspects of the lagoons’ ecosystem services. Another challenge is how to implement the needed changes (see Fig. 4) in these times of uncertainty with regard to the economy and environmental change. For instance, for all lagoons, independently of geographical location, hydrology characteristics or/and governance issues, there is the need to improve the monitoring networks as part of Research, Technology and Development (RTD) activities (Fig. 4). Monitoring programmes are also essential *Responses* (*sensu* DPSIR) of EU Directives^50^,^51^. The major challenge is to do this in a cost-effective way, taking into account the common links within lagoons ecosystems and at the same time considering their specificities. The aim is to better allocate efforts and economic resources to prioritize lagoon conservation goals in an effective manner, taking into account the country social-economic needs and acknowledging that the different lagoon’s stakeholders might have different perceptions on the importance of their natural resources^11^,^52^ and on the maintenance of specific ecosystem services.

*Drivers* are inter-connected, as clarified in the mosaic-DPSIR, so changes in the DPSIR of one *driver* will affect the outcome of another. This way, it is important to consider *drivers* that have the potential to grow in a sustainable way, the ones that can be managed to reduce negative ecological *impacts* without compromising the economy, and also to consider the transversal *drivers* and the *natural change* that might affect the *state* of all anthropogenic *drivers.* All of these are interconnected. For instance, a unique local management structure (Fig. 4) would enable us to concentrate efforts and in this way to save economic resources so as to able to allocate them in other areas. However, a unique management structure might be a challenging task for all lagoons, and particularly for the ones with transboundary management. Another example is sustainable tourism, which is a *driver* with the potential to grow, attract investment, generate employment and create wealth^53^. It also aligns with the EU’s growth strategy for a smart, sustainable and inclusive EU economy within the Europe 2020 (COM(2014) 85 final, 2014/0044). Together with efforts to raise stakeholders’ civic and environmental awareness, sustainable tourism could help to solve the problem of desertification in some regions of the lagoon (e.g. in Tyligulskyi Liman Lagoon) and to avoid overexploitation of resources in the present context of unemployment and economic crisis.

## Conclusions

In this study, we have demonstrated a decision-support framework for coastal lagoons management grounded in four case-study lagoons. The methodology is a further development of the classical DPSIR-approach, where we have tried to integrate *drivers* into a mosaic-DPSIR conceptual model. The underlying concept has its basis in sustainability principles and in an integrative and adaptive management of the lagoons uses, where we have: 1) addressed the human needs, by selecting the main *drivers* in the lagoons and needs for development as identified from the lagoons’ stakeholders, 2) contributed to the understanding of the *impacts* of those *drivers*, taking into account ecosystem resources, where *impacts* were evaluated considering the ecosystem services provided by each lagoon. In this way we have contributed to an understanding of the outcomes of a *driver* both from an ecological and socio-economic view in order to structure policy-relevant options; 3) acknowledged the interacting ecosystem uses through the mosaic-DPSIR approach; 4) proposed integrated recommendations for management, taking into account the reference *state*, the multiplicity and the interdependency among the different ecosystem uses. The information used in the mosaic-DPSIR was obtained from an integrated science-stakeholder interface, as a basis for a proactive management. Within a Pan-European context, we were able to propose common recommendations for the management of European lagoons, based on the current problems and opportunities of the four lagoons used in this study. It is however important to consider the specificities of each lagoon and the fact that different policy options might impact ecosystem services and the decision makers themselves, meaning that regional and local management has to take into account the context of the coastal system specific needs.

## Additional Information

**How to cite this article**: Dolbeth, M. *et al*. An integrated Pan-European perspective on coastal Lagoons management through a mosaic-DPSIR approach. *Sci. Rep.* **6**, 19400; doi: 10.1038/srep19400 (2016).

## Supplementary Material

Supplementary Information

## Figures and Tables

**Table 1:** Characteristics of the case-study lagoons.

	Ria de Aveiro	Mar Menor	Tyligulskyi Liman	Vistula Lagoon
Countries	Portugal	Spain	Ukraine	Poland/Russia
Lagoon area (km^2^)	83	135	129	838
Catchment area (km^2^)	3556	1380	5240	20 730
Sea/Ocean	Atlantic Ocean	Mediterranean sea	Black Sea	Baltic Sea
Main tributaries	Vouga	Albujon	Tyligul	Pregola Pasleka Elblag
Total freshwater inflow (km^3^year^−1^)	2.14	0.009	0.023	3.69
Salinity range	0–36	42–47	15–23	1–7
Average temperature (°C)	14	25	9.7	7.7
Average precipitation and range (mm year^−1^)	1100 (600–2100)	337 (300–370)	515 (470–570)	750 (670–860)
Major land use	Agriculture (29%); Forest (56%)	Agriculture (82%); Forest (1%)	Agriculture (80%); Forest (4%)	Agriculture (67%); Forest (25%)

**Table 2:** Identified anthropogenic *drivers* in each lagoon case study.

	Ria de Aveiro	Mar Menor	Tyligulskyi Liman	Vistula Lagoon
Population density and growth	✓	✓	✓	✓
Tourism and related activities	✓ (e.g. sports and recreational activities, hunting, traditional festivals)	✓ (e.g. intensive beach tourism, golf and recreational activities)	✓ (e.g. sustainable tourism in the regional landscape parks)	✓ (e.g. beach tourism*)
Harvesting activities	✓ Agriculture (maize and wheat), Livestock (e.g. Marinhoa breed), Aquaculture (e.g. seabass, seabream, turbot, Pacific oyster, European clam)	✓ Intensively irrigated agriculture (e.g. horticultural); Livestock (e.g. pig)	✓ Agriculture (e.g. cereals, vegetables, cucurbitaceous viticulture); Livestock (e.g. poultry)	✓ Agriculture (e.g. cereals, potato)
Natural resources demands	✓ Fish (e.g. seabass, seabream, lamprey); Shellfish (e.g. cockles, oysters, shrimps, crabs); and Bait (e.g. “*casulo*”-*Diopatra neapolitana, Hediste diversicolor*) catches	✓ Fish (e.g. Sparidae & Mugilidae species, eel); and Shellfish (e.g. prawns) catches	✓ Fish catches* (e.g. Gobiidae, Atherinidae species)	✓ Fish catches (e.g. herring, pikeperch, eel)
Connection to sea infrastructure and maritime transport	✓ Harbour, port facilities and maritime transport	✓ Harbour, port facilities and maritime transport	✓ Artificial channel	✓ Harbour, Maritime transport
Industry and related activities (mining extraction included)	✓ Industry and industrial historical contamination (restricted to 2 km^2^ basin)	✓ Mining historical contamination (restricted to southern area)	Not relevant	✓ Industry*
Uncoordinated management/ Insufficient transboundary cooperation	✓ Uncoordinated management	✓ Uncoordinated management	✓ Uncoordinated management and lack of regulatory plans	✓ Insufficient transboundary cooperation and uncoordinated management
Economic crisis	✓	✓	✓	✓

Detailed DPSIR cycles of the most important *drivers* are available in supplementary information.

^*^Drivers identified in the lagoon, but with little expression with regard to the economy and ecological *impacts.*

**Table 3:** Natural changes identified in each lagoon case study, potentially affecting the *state change*.

	Ria de Aveiro	Mar Menor	Tyligulskyi Liman	Vistula Lagoon
Natural characteristics of the lagoon (ecohydrology, geomorphology, etc)	✓	✓	✓	✓
Invasive species	✓ Several species (e.g. bivalves *Venerupis philippinarum, Corbicula fluminea,* plant *Eichhornia crassipes*)	✓ (e.g. gastropod *Hexaplex trunculus)*	✓ (e.g. polychaete *Polydora cornuta*)	✓ Several species (e.g. polychaete *Marenzelleria,* bivalve *Rangia cuneata*)
Unbalanced natural biological populations	✓ Cormorants, storks	✓ Macroalgae (*Caulerpa prolifera*), Jellyfish species		✓ Cormorants
Coastal erosion	✓			✓
Extreme weather events*	✓ e.g. heat waves, heavy rainy periods, droughts, storm surge	✓ e.g. heat waves, droughts	✓ e.g. heat waves, heavy rainy periods, droughts	✓ e.g. heat waves, heavy rainy periods, droughts
Global climate change* (e.g. sea-level rise, global warming, altered patterns of precipitation)	✓	✓	✓	✓

Local management cannot address the causes of change, only the consequences.

^*^We defined global climate change as future change trends in weather patterns that last over a significant period of time and occur globally, whereas extreme weather events are defined as severe or unseasonal weather events occurring nowadays.
