# Supplementary material for: An integrated Pan-European perspective on coastal Lagoons management through a mosaic-DPSIR approach
Source: Sci Rep. 2016 Jan 18;6:19400. doi: 10.1038/srep19400 (PMC4725967; doi:10.1038/srep19400)
Supplement: Supplementary Information [file srep19400-s1.pdf]

Supplementary information

An integrated Pan-European perspective on coastal Lagoons management through a mosaic-DPSIR approach

Marina Dolbeth[1,2*], Per Stålnacke[3], Fátima L. Alves[4], Lisa P. Sousa[4], Geoffrey D Gooch[5], Valeriy Khokhlov[6], Yurii Tuchkovenko[7], Javier Lloret[8,9], Małgorzata Bielecka[10], Grzegorz Rozynski[11], João A. Soares[4], Susan Baggett[5], Piotr Margonski[12], Boris V. Chubarenko[13], Ana I. Lillebø[1*]

[1] Biology Department & Centre for Environmental and Marine Studies (CESAM), University of Aveiro, Campus Universitário de Santiago, 3810-193 Aveiro Portugal
[2] CFE – Centre for Functional Ecology, Department of Life Sciences, University of Coimbra, PO Box 3046, 3001-401 Coimbra, Portugal
[3] Bioforsk - Norwegian Institute for Agricultural and Environmental Research, Ås, Norway
[4] Department of Environment and Planning & Centre for Environmental and Marine Studies (CESAM), University of Aveiro, Campus Universitário de Santiago, 3810-193 Aveiro Portugal
[5] Dundee Centre for Water Law, Policy and Science, University of Dundee, Dundee, Scotland, UK
[6] Department of Theoretical Meteorology and Weather Forecasts, Odessa State Environmental University, Odessa, Ukraine
[7] Department of Oceanology and Marine Nature Management, Odessa State Environmental University, Odessa, Ukraine
[8] Department of Ecology and Hydrology, University of Murcia, Murcia, Spain
[9] The Ecosystems Center, Marine Biological Laboratory, Woods Hole MA US 02543
[10] Department of Wave Mechanics and Dynamics of Structures. Institute of Hydro-Engineering Polish Academy of Sciences, Gdansk, Poland
[11] Department of Coastal Engineering and Dynamics, Institute of Hydro-Engineering Polish Academy of Sciences, Gdansk, Poland
[12] National Marine Fisheries Research Institute, Gdynia, Poland
[13] Atlantic Branch of P.P.Shirshov Institute of Oceanology of Russian Academy of Sciences, Kaliningrad, Russia

DPSIR framework application to each case study

Introductory note

The DPSIR framework describes the interactions between society and the environment, allowing to structure and communicate policy-relevant research: D - drivers, P - pressures, S - state change, I - impact, R – responses [1]. A mosaic-DPSIR is an

adapted framework, where DPSIR cycles from several drivers in an environmental system are combined, may have interactions among each other, and, as such, need integrated management recommendations (responses) [1].

In this study, we applied the mosaic-DPSIR for each case study lagoon. Initially, DPSIR cycles of the anthropogenic drivers considered as most important for each lagoon were done (Table 1 from the paper), by combining information from different sources: from the deliverables and technical briefs from LAGOONS project on the current knowledge base and knowledge gaps, problem based sciences analysis and references therein (WP2, WP3, WP7), focus groups and citizen jury briefs (WP4) with information from the lagoons' end users (stakeholders, policy makers, general public) (see e.g. in [2]) and on the expert judgement from the LAGOONS different team members. All documentation is available in [3] and in the LAGOONS website, http://lagoons.biologiaatua.net. However, some briefs are only available in the native language (e.g. focus group and citizen jury briefs).

Next, all DPSIR cycles and mosaic-DPSIR are presented for each lagoon: Ria de Aveiro, Mar Menor Lagoon, Tyligulskyi Liman Lagoon, and Vistula Lagoon. In the *Responses*, common general responses among cycles are marked in bold.

Common references

1. Atkins, J. P., Burdon, D., Elliott, M. & Gregory, A. J. Management of the marine environment: Integrating ecosystem services and societal benefits with the DPSIR framework in a systems approach. *Mar Poll Bull* **62,** 215–226 (2011).
2. Sousa, L. P., Lillebø, A. I., Gooch, G. D., Soares, J. A. & Alves, F. L. Incorporation of Local Knowledge in the Identification of Ria de Aveiro Lagoon Ecosystem Services (Portugal). *J Coast Res* **65,** 1051–1056 (2013).
3. Lillebø, A. I., Stålnacke, P. & Gooch, G. D. *Coastal Lagoons in Europe: Integrated Water Resource Strategies*. (IWA Publishing) (2015, forthcoming).

Ria de Aveiro

A

Drivers (Ria de Aveiro):
Population, tourism and related activities

Pressures:
High urban/touristic growth in coastal areas and expansion to areas with great ecological value;
Misuse of infrastructure and lack of users' awareness;
Excessive speed of motor boats and jet skis;
Recreational hunting duck-resources;
Beach tourism, sport activities.

State change:
Great number of habitats supporting great biological diversity and several protected areas, habitats and species;
Abandonment of traditional activities (degradation of salt pans);
Classified as reasonable good state of environmental preservation (only 2 areas were con-sidered vulnerable with regard to eutrophication);
Possible lead contamination in specific water line (hunting);
Great offer for sport and recreational activities (kitesurf, diving, sailing, biking, tracking);
São Jacinto population isolation associated to deficiency in lagoonal transport.

Positive Impacts:
Several attractions for different tourism sectors (ecotourism, recreational hunting, sports, traditional festivals – e.g. "*moliceiros*" race, eel and Aveiro sweet-eggs, codfish, lamprey, among other festivals;
Employment, wealth creation (local economy stimulated);
Sustainable touristic activities have potential to growth;
Ria products are elements of local cultural identity (e.g. salt, fish and shellfish resources).

Negative Impacts:
Salt-works and "*moliço*" collection are declining activities, with low economic expression;
Increase of motorboats and jet skis may cause imbalance in the system;
Lack of investment in tourism;
Lack of information and promotion of Ria as a whole, with information and activities mostly restricted to central channels of Aveiro.

Responses:
Better overall regulation, law enforcement: Improve surveillance and law enforce-ment (e.g. recreational fishing, hunting resources and their habitats); Better manage-ment of touristic activities.
Increase civic awareness and improve local participation
Promote high-end, sustainable tourism: Promote ecotourism, gastronomic tourism; Better overall promotion of area as a whole and product labelling (e.g. salt); Support traditional activities (traditional boats "*moliceiros*", salt production, reeds and "*moliço*" collection); Create intermodal connections to promote easy and fast transportation by e.g. train, boat and bike hire; Encourage elite tourism in the region.
Improve or create new infrastructure: Infrastructure to improve tourism offer for beach recreation, transport, parking areas;
RTD activities, improve knowledge to reduce uncertainty: Monitor lead in specific water lines to evaluate the origin: industrial or from hunting.

B

Drivers (Ria de Aveiro)
Agriculture, Livestock, Aquaculture

Pressures:
Fragmented landscape (traditional smallholdings and impoverish-ment of the salt pan landscape);
Water needs/supply;
Conflict of interest between these drivers and others (harbour and port related):
Diffuse pollution: nutrient loading;
Point source pollution: nutrients (<10%), contaminants of poten-tial concern and emergent pollutants;
Possible introduction of pathogenic agents in the bypass of oys-ter seeds, non-indigenous species (e.g. *Ruditapes philippina-rum*) and fish juveniles from nurseries to aquaculture;
Economic value of the drivers.

State change:
Site-specific vulnerable areas with regard to nutrient concentration;
Changes in water quality inside the lagoon (spatial variability);
Site-specific increase of biodiversity – "*bocage*" green infrastructure;
Presence of biotoxins;
Increased primary sector activity for additional income;
Presence of certified Marinhoa breed (indigenous cattle species in the Baixo Vouga Lagunar marsh area);
Decline of rice fields (small traditional rice wetlands);
Proliferation of non-indigenous species in the lagoon (e.g. *Ruditapes phillippinarum*).

Positive Impacts:
Employment; Wealth creation (improve local and national economy, exportations);
Supports other economic activities (e.g. preparation and processing industry, storage, transport and distribution, marketing, business support services);
Additional income;
Higher habitat complexity with associated biodiversity increase (amphibian, reptile, otter, …);

Negative Impacts:
Habitat loss (e.g. saltpans - valuable habitats for waders);
Decrease potential for ecotourism and cultural heritage (decreased traditional activities and saltpans conversion);
Temporarily bivalves harvesting interdicted due to biotoxins;
Surface runoff of nutrients, contaminants of potential concern and emergent pollutants:
Lack of strategic planning with regard to the drivers;
Biodiversity loss associated to the rice field wetlands;
Competition between native and non-indigenous clam species (e.g. *Ruditapes decussatus* vs *R. philippinarum*).

Responses:
Better overall regulation, law enforcement: Improve surveillance to apply legislation and minimize negative impacts in the environment; Contribute to implement WFD and other legislation in vigour;
Unique local management structure: Integrate and standardize management plans;
Increase civic and environmental awareness: Improve local participation, engage different stakeholders and representatives of other drivers - fishermen, aquaculture, agriculture and port interests;
RTD activities, improve knowledge to reduce uncertainty: Consider vulnerable areas for specific monitoring programs and management measures; Improve the procedures to monitor biotoxins;
Optimization of water use: Improve wastewater treatment plants; Implement programs for a sustainable use of water resources.
Improve or create new infrastructure: Complete the Baixo Vouga dike.
Restoration measures: Restore and clean the small drainage channels of "*Baixo Vouga Lagunar*"

C

Drivers (Ria de Aveiro):
Harbour, port and maritime transport

Pressures:
Dredging activities (navigation channels);
Construction of inlet piers;
Marginal retentions;
Breakwater construction;
Silted up areas;
Alteration in sediments dynamics (erosion and deposition dynamics);
Conflict of interest between this driver and others (fishermen, aquaculture, agriculture and port interests).

State change:
Changes in the system's hydrodynamics: increases in tidal water velocity, water turbidity and channel depth, changes in tidal prism and light penetration;
Alterations of water physicochemical characteristics;
Decline in intertidal area, mudflats, seagrass meadows and salt-marshes;
Nutrient cycling modified related to loss of seagrasses;
Increase of the saltwater flooding period, surface slat water intrusion, salinization of agricultural areas (e.g. baixo vouga);
Impoverishment of sediments bed and modifications in sediment dynamics.

Positive Impacts:
Employment, wealth creation;
Supports other economic activities (e.g. industry, including fish processing, storage, transport and distribution, marketing, business support services);
Maritime traffic.
Negative Impacts:
Habitat loss and fragmentation (e.g. saltmarsh, seagrass, typical landscape -"*bocage*");
Biodiversity decrease (biological resources dependent on saltmarsh and seagrass);
Decreased protection against erosion;
Changes in ecosystem functioning (decrease settling of fine sediments, decrease nutrient retention;
Collapse of abandoned salt-works;
Salinization of land: Loss of agriculture land and ecological succession to halophytes plants.

Responses:
Better overall regulation, law enforcement: Contribute to implement WFD; Settlement of plan for dredging and its surveillance; Revise the current legislation;
Unique local management structure: Integrate management plans and coordinate the management of the human activities in the lagoon;
Increase civic awareness and improve local participation: engage different stakeholders and representatives of the drivers - fishermen, aquaculture, agriculture and port (e.g. Polis Litoral, Farming association, Coastal Action Group, Intermunicipal Community, Association of Producers and Marnotos, Natural Reserve of Dunes of S. Jacinto);
RTD activities, improve knowledge to reduce uncertainty: develop a decision-support tool for management strategies, taking into account biolo-gical, water quality and hydrodynamic models of the intertidal seagrass communities;
Improve or create new infrastructure: sustainable infrastructure for maritime transportation and to control currents and water velocity;
Improve the lagoon's navigability: e.g. channels' navigability.

D

Drivers (Ria de Aveiro)
Fish, shellfish and bait catches

Pressures:
Professional and recreational fishing, bait digging
Increased pressure on communities of fish, shellfish and bait - dwindling stocks
Unreported catches, sold directly to restaurants
Illegal fishing gears
Lack of vigilance (especially recreational fishing) and lack of punishment for defaulters (illegal fishing)
Conflict of interest between these drivers and others (port, harbour, maritime transport)
Economic value of the drivers

State change:
Fish, shellfish and bait decrease (fishing pressure and illegal activities)
Impoverishment of sediments bed
Non-target species decrease (due to illegal trawling e.g. cockle, clams)
Increase of younger and older people looking for additional income and increased number of women due to unemployment associated to economic crisis

Positive Impacts:
Employment, wealth creation (local and national economy);
Local socio- cultural identity;
Additional income to overcome economic difficulties and unemployment
Supports other economic activities (e.g. fish, shellfish processing industry, storage, transport and distribution, marketing, business support services)
Attraction for tourists (recreational fishing).
Negative Impacts:
Parallel economy (unreported catches);
Overall biodiversity decrease;
Commercial species decline;
Potential overfishing cannot be ascertained;
Loss of important ecological processes and functions (e.g. bioturbation, ecosystem engineering);
Potential increase of non-licensed catches and gears to overcome economic crisis.

Responses:
Better overall regulation, law enforcement: Regulate catches; Improve surveillance and law enforcement for illegal fishing gears, unreported catches, recreational fishing); Target species quota restrictions related to quantity, size at catch and compliance with shellfishing ban periods;
Unique local management structure: Integrate and standardization management plans;
Increase civic and environmental awareness: Improve local participation; Engage different stakeholders and representatives of the other drivers - fishermen, aquaculture, agriculture and port interests;
RTD activities, improvement of knowledge to reduce uncertainty: Specific monitoring programmes and management measures for vulnerable areas; Create protected breeding areas within lagoon; Implement studies on the lagoon's carrying capacity and socio-economic importance for the driver and improve the procedures related to the monitoring of biotoxins.

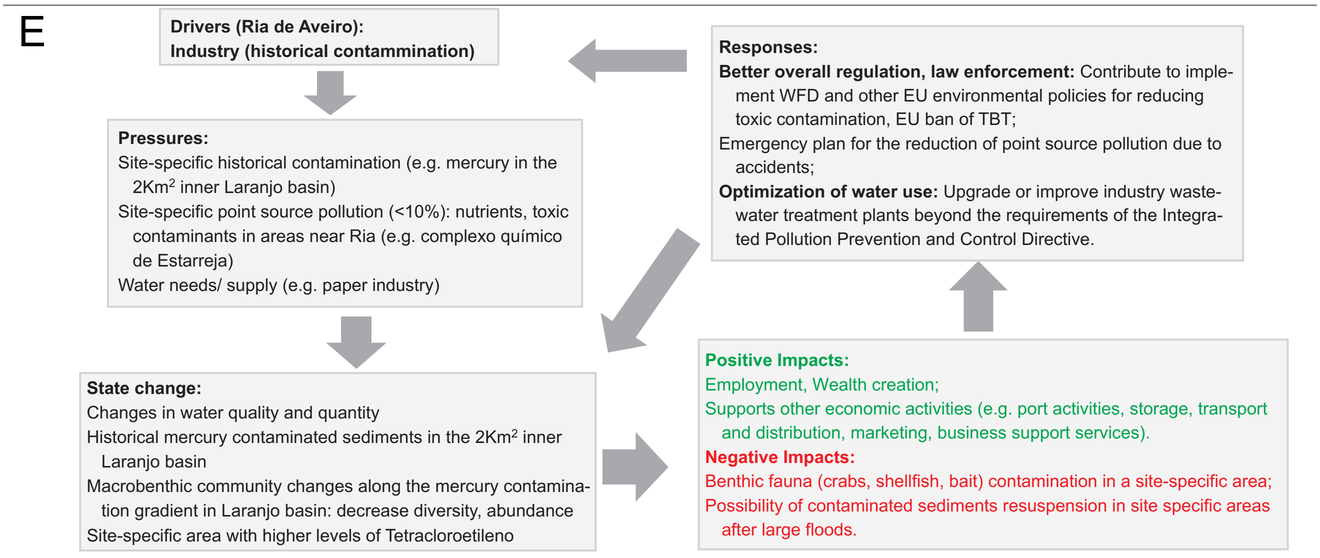

Supplementary Figure S1. DPSIR cycles for the *drivers* of Ria de Aveiro. A) Population, tourism and related activities; B) Aquaculture, agriculture and livestock; C) Harbour, port and maritime transport; D) Fish, shellfish and bait catches; E) Historical contamination associated to the mercury industry.

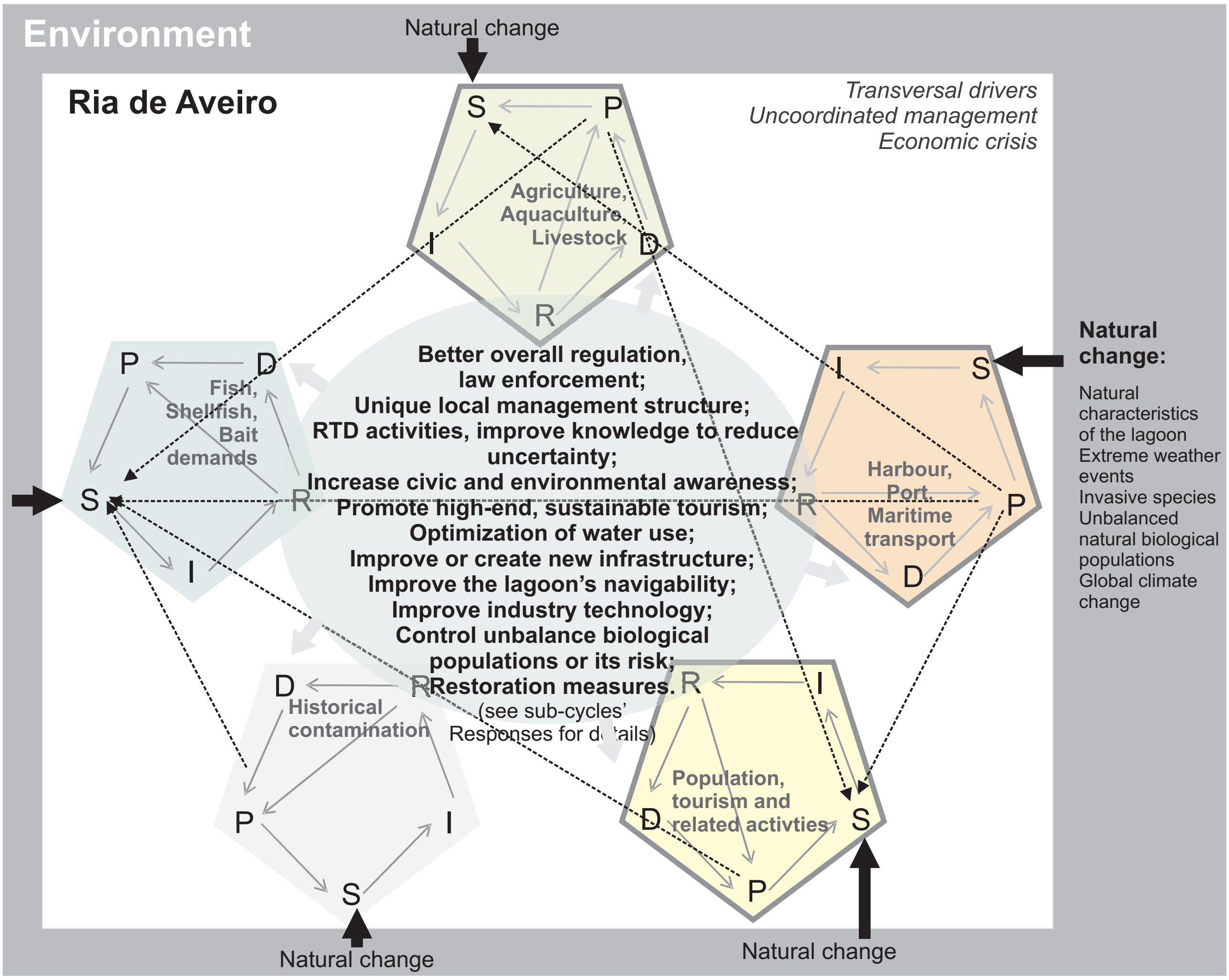

Supplementary Figure S2. Mosaic-DPSIR for Ria de Aveiro, with emphasis on the *drivers* with higher social-economical expression in the lagoon, showing interactions among DPSIR cycles (dotted arrows), the influence of the natural change on the *state* (black arrows) and that *responses* from each cycle should be combined into common integrated *responses.*

DPSIR data from Ria de Aveiro available in the following references:

Azevedo, A., *et al.*, Application of the generic DPSIR framework to seagrass communities of Ria de Aveiro: a better understanding of this coastal lagoon. *J Coast Res* **65**, 19–24 (2013)

LAGOONS. The Ria de Aveiro Lagoon - Current knowledge base and knowledge gaps. LAGOONS Report D2.1b. 52pp. (2012) Available in http://lagoons.biologiaatua.net

LAGOONS. Results of the problem based science analysis: The Ria de Aveiro Lagoon. *LAGOONS Report D3.2.1.* 50 pp. (2013) Available in http://lagoons.biologiaatua.net

LAGOONS. Grupos de Discussão - Ria de Aveiro, Portugal. LAGOONS Destaque B4.1. 4pp. (2013) Available in http://lagoons.biologiaatua.net (in Portuguese)

LAGOONS. Painel de Cidadãos - Ria de Aveiro, Portugal. *LAGOONS Destaque B4.2.* 3pp. (2013) Available in http://lagoons.biologiaatua.net (in Portuguese)

LAGOONS. Activities report: Report on raising public participation and awareness including design of uptake and capacity building activities. *LAGOONS Report D4.1*, 61pp. (2014). Available in http://lagoons.biologiaatua.net

Lillebø, A. I., *et al.* The Physio-geographical background and the Ecology of Ria de Aveiro. In Coastal Lagoons in Europe: Integrated Water Resource Strategies (Lillebø, A. I., Stalnacke, P. & Gooch, G. D.) (IWA Publishing, 2015)

PGBH Plano de Gestão das Bacias Hidrográficas do Vouga, Mondego e Lis. Parte A – Avaliação Ambiental Estratégica. Relatório Ambiental, 445 pp. (2012a)

PGBH Plano de Gestão das Bacias Hidrográficas do Vouga, Mondego e Lis. Parte 2 – Caracterização Geral e Diagnóstico. 6.2 – Relações entre o estado e as pressões que são responsáveis por este estado. (2012b)

Sousa, L. P., *et al.*. Incorporation of Local Knowledge in the Identification of Ria de Aveiro Lagoon Ecosystem Services (Portugal). *J Coast Res* **65,** 1051–1056 (2013).

Sousa, L. P., *et al*. The Management Story of Ria de Aveiro. In Coastal Lagoons in Europe: Integrated Water Resource Strategies (Lillebø, A. I., Stalnacke, P. & Gooch, G. D.) (IWA Publishing, 2015).

Mar Menor

A

Drivers (Mar Menor):
Population, tourism and related activities

Pressures:
High urban/tourism growth - Invasion of the marine-land public domain;
Degradation of landscape and protected areas;
Seasonality - high population density in summer (10-fold↗);
Urban wastewater effluents (untreated or insufficiently treated water);
Diffuse pollution and point source pollution;
Increased water sport activities and number of boats;
Increasing water needs;
Sand replenishment of beaches.

State change:
Eutrophication: great amounts of nitrate, phosphate and ammonium, especially in summer in water and sediments - overall water quality decrease;
Presence of waste;
Acoustic and light pollution;
Loss and fragmentation of natural habitats of high ecological values;
Spread of macroalgae *Caulerpa prolifera* / Replacement of seagrass by the algae;
Allocthonous jellyfish blooms;
Changes in infiltration/runoff process in the basin;
Changes in sediment dynamics.

Positive Impacts:
Employment, wealth creation (local economy stimulated);
Sustainable tourism activities have potential to growth, i.e. great number of habitats and landscape supporting biological diversity; several protected areas and species;
Safe beaches with no dangers around; traditional activities (salt-works).

Negative Impacts:
Overcrowding due to boosted development;
Loss of area for croplands and fertile soils;
Biodiversity decline;
Protected natural areas and habitats threatened;
Disturbance to nesting, migration and wintering of aquatic bird populations;
Problems with waste management, wastewater and traffic;
Degradation of water quality and ecological condition of the lagoon;
Increased risk of natural disasters (e.g. flash-flood of greater intensity);
Damage to dune systems and extensive beaches;
Habitat loss and the lagoon's completion by sand replenishment.

Responses:
Better overall regulation, law enforcement: better management of urban development, boats, waste management.
Promote high-end, sustainable tourism: ecotourism, traditional activities, stimulate traditional fishing arts.
Increase civic and environmental awareness: promote environmental education.
RTD activities, improve knowledge to reduce uncertainty: Assess the lagoon's response to environmental stressors and resilience to eutrophication impacts using lagoon's resources (jellyfish);
Unique local management structure: Standardize management plans and responsible entities (e.g. jurisdiction limits for different authorities are not clear enough).

B

Drivers (Mar Menor)
Agriculture and livestock:

Pressures:
Intensively irrigated agriculture in watershed area (>80%);
Increased water use/supply (Campo de Cartagena): water extraction from aquifers, Tajo-Segura river diversion;
Changes in water balance;
Diffuse pollution: nutrient loading (mainly nitrate), contaminants of potential concern (pesticides);
Agricultural drains into wetlands;
Soil erosion.

State change:
Eutrophication;
Primary production increase by macroalgae *Caulerpa prolifera* and decline of macrophyte *Cymodocea nodosa;*
Seasonal allocthonous jellyfish blooms;
Site-specific area with sediment deterioration (OM accumulation, anoxia, fish decrease);
Habitat loss and fragmentation (e.g. saline steppe);
Groundwater overexploitation;
Rise in superficial phreatic levels;
Increased water flows into the wetlands;
Site-specific groundwater contamination.

Positive Impacts:
Employment and wealth creation (Campo de Cartagena is one of the most productive and profitable agricultural areas in Europe).

Negative Impacts:
Decline of macrophyte *Cymodocea nodosa;*
Loss of natural biodiversity;
Degradation of water quality and ecological condition in the lagoon;
Potential negative impacts on tourism and fishing;
Habitat loss of natural habitats with high ecological value (saline steppe);
Natural protected areas threatened.
Marine water intrusion into the aquifers (drought).

Responses:
Optimization of water use: Implement programmes for a sustainable use of water resources; Improve wastewater treatment plants.
Better overall regulation, law enforcement: Better management of human activities; Contribute to implement WFD.
RTD activities, improve knowledge to reduce uncertainty: Assess the lagoon's response to envi-ronmental stressors and resilience to eutrophication impacts using lagoon's resources (jellyfish).
Restoration measures: need for rehabilitation measures.
Unique local management structure: Integrate and standardize management plans.
Agriculture production and technologies: Improve agricultural technology and practices to reduce the amount of pollutants reaching the groundwater; Change agriculture production systems (dry crops and/or highly profitable crops with lower water requirements).
Increase civic and environmental awareness: Increase public & end-users awareness; Improve local participation.

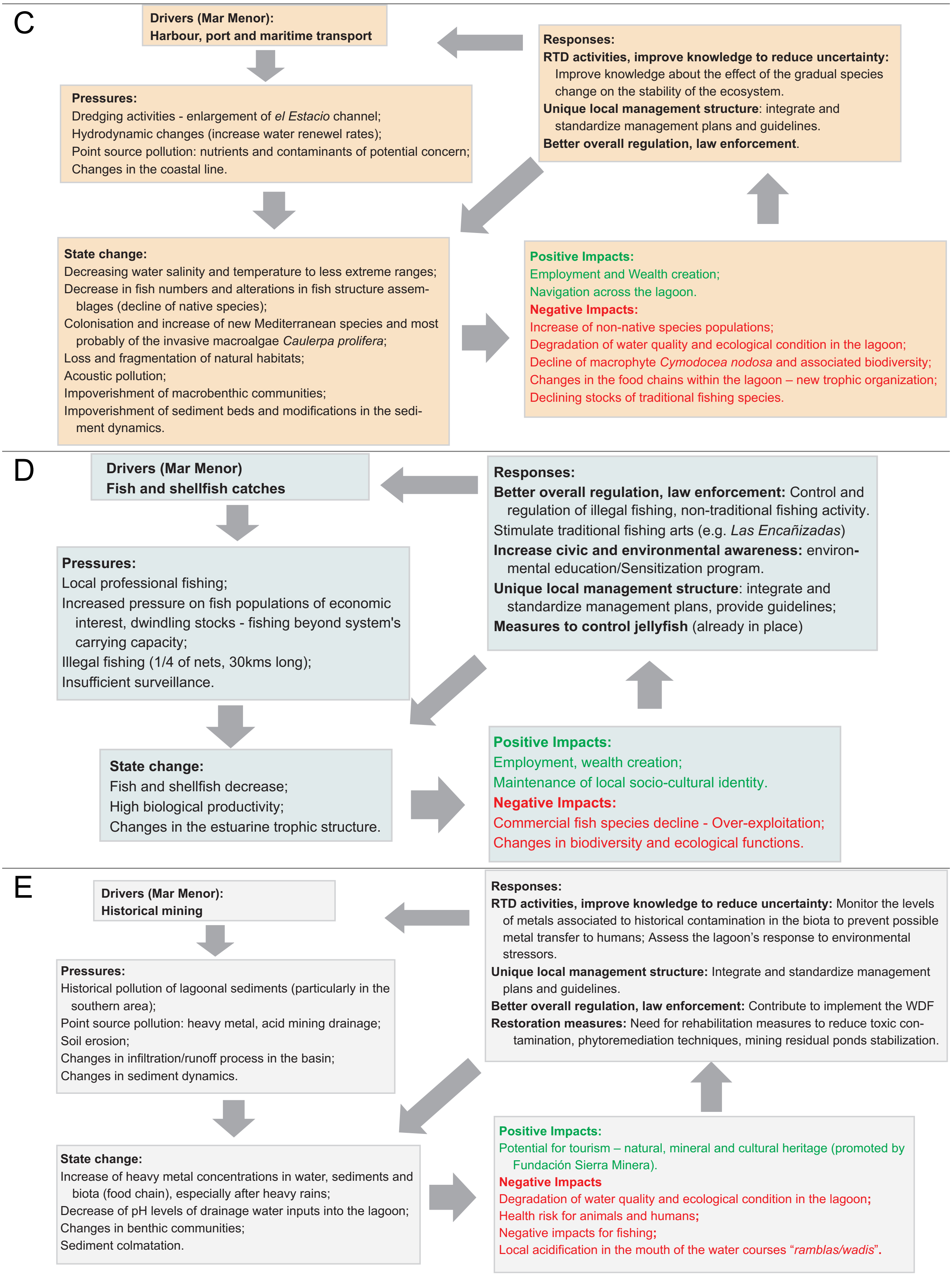

Supplementary Figure S3. DPSIR cycles for the *drivers* in Mar Menor. A) Population, tourism and related activities; B) Agriculture and livestock; C) Harbour, port and maritime transport; D) Fish and shellfish catches; E) Historical mining (e.g. lead, zinc and iron).

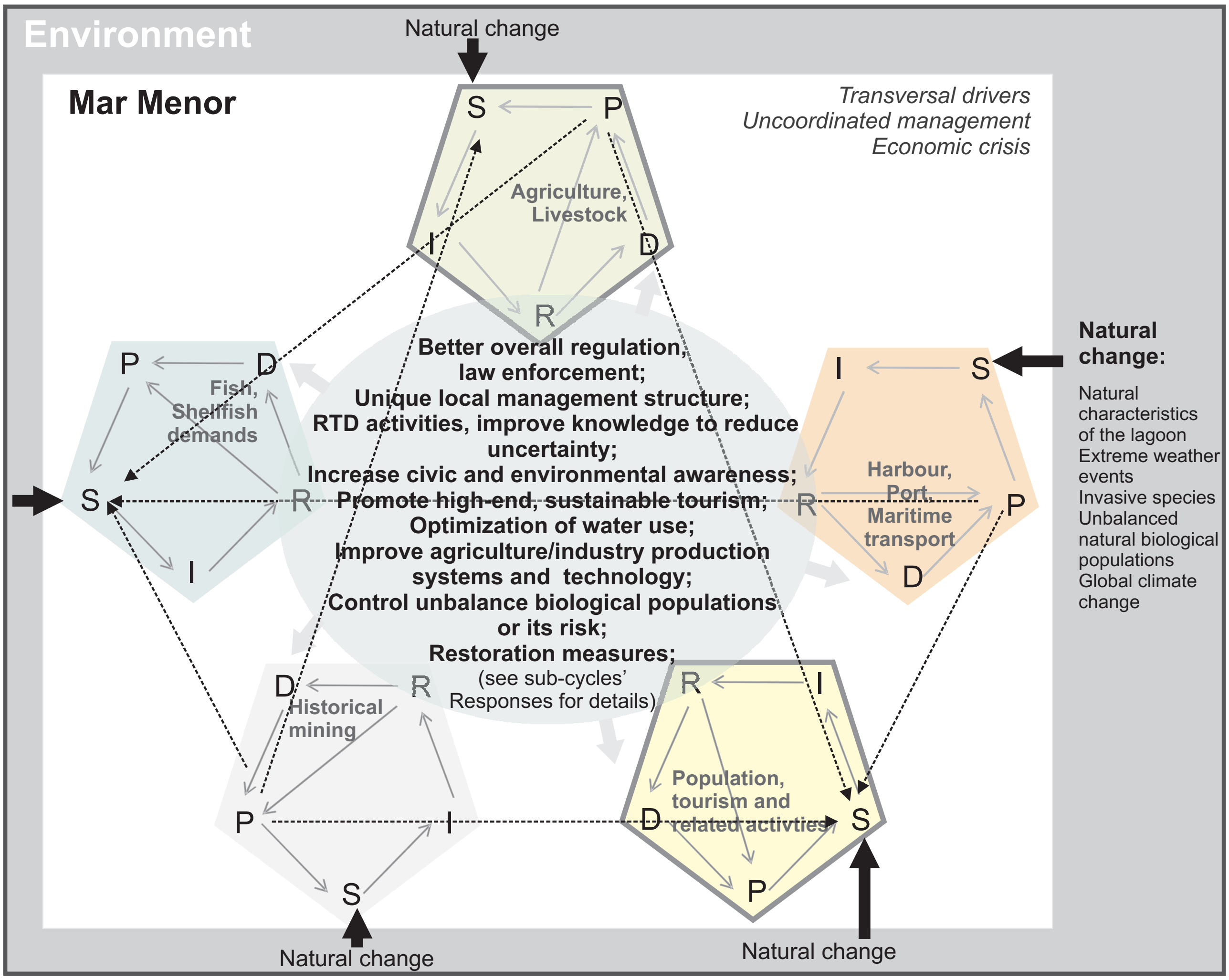

Supplementary Figure S4. Mosaic-DPSIR for Mar Menor, with emphasis on the *drivers* with higher social-economical expression in the lagoon, showing interactions among DPSIR cycles (dotted arrows), the influence of the natural change on the *state* (black arrows) and that *responses* from each cycle should be combined into common integrated *responses.*

DPSIR data from Mar Menor available in the following references:

LAGOONS. The Mar Menor - Current knowledge base and knowledge gaps. *LAGOONS Report D2.1c.* 65pp. (2012) Available in http://lagoons.biologiaatua.net

LAGOONS The Mar Menor Lagoon, Spain. LAGOONS Technical Brief TB3. 5pp. (2012) Available in http://lagoons.biologiaatua.net

LAGOONS. Results of the problem based science analysis: The Mar Menor. *LAGOONS Report D3.2.2.* 50 pp. (2013) Available in http://lagoons.biologiaatua.net

LAGOONS. Grupos de Discusión – Mar Menor, España. LAGOONS Resumen B4.1. 4pp. (2013) Available in http://lagoons.biologiaatua.net (in Spanish)

LAGOONS. Activities report: Report on raising public participation and awareness including design of uptake and capacity building activities. *LAGOONS Report D4.1*, 61pp. (2014). Available in http://lagoons.biologiaatua.net

Lloret J., *et al*.,. The Management Story of Mar Menor. In Coastal Lagoons in Europe: Integrated Water Resource Strategies (Lillebø, A. I., Stalnacke, P. & Gooch, G. D.) (IWA Publishing, 2015)

Marín A., *et al*.,The Physio-geographical Story of Mar Menor. In Coastal Lagoons in Europe: Integrated Water Resource Strategies (Lillebø, A. I., Stalnacke, P. & Gooch, G. D.) (IWA Publishing, 2015)

Tyligulsky Liman Lagoon

A

Drivers (Tyligulsky):
Population, tourism and related activities

Pressures:
Urban/tourism growth (e.g. resorts, cottages);
Seasonality - high population density in summer;
Unregulated touristic activities;
Unregulated development of cottage areas, not connected to sewage systems: untreated wastewater effluents;
Degradation of landscape and disturbance of protected areas;
Illegal sand extraction/excavation;
Increase of landfills garbage dumps;
Fire occurrence;
Point source and diffuse pollution: nutrients and household rubbish;
Illegal hunting and fishing (pouching);
Increased water needs;
Competing views and disagreement regarding Landscape Park;
Lack of information signs (for protected areas).

State change:
Eutrophication (seasonal and site-specific areas): hypoxia, periodic fish and hydrobionts kills, reduced water transparency, phytoplankton blooms during summer;
Presence of waste;
Impoverishment of flora and fauna - e.g. population decline of duck and other avifauna;
Loss and fragmentation of natural habitats of great ecological values.

Positive Impacts:
Employment, wealth creation (local economy stimulated);
Sustainable touristic activities have potential to growth - great number of habitats and landscape supporting biological diversity; several protected areas and species; therapeutic activities (mud baths);
Negative Impacts:
Biodiversity decline (e.g. avifauna);
Lack of funds to invest;
Degradation of water quality and ecological conditions in the lagoon;
Competing views and disagreement regarding Landscape Park;
Problems with waste management, wastewater treatments;
Protected natural areas and habitats threatened.

Responses:
Promote high-end, sustainable tourism: Promote ecotourism, Nature-based tourism (e.g. ornithology), therapeutic (mud therapy).
Increase civic and environmental awareness: Promote environmental education and improve local participation.
Optimization of water use: Limit and regulate freshwater withdrawal for filling of ponds and reservoirs at Tyligul river and lagoon drainage basin.
Better overall regulation, law enforcement: Control the agricultural activity at homestead land; Develop, control and regulate touristic activities; Regulate and legislate hunting and fishing activities; Implement the requirements of environmental legislation.
Improve or create new infrastructure: Increase investment in the Park (e.g. information signs for protected areas, stone lined fireplaces, eco-tourism guides and trails, information on region's protected areas, health resorts for balneology and therapeutic mud baths; Create a unified system for sewage.
Unique local management structure: Integrate and standardize management plans and responsible entities for sustainable development of the area; Better inter-region regulation.

B

Drivers (Tyligulsky):
Agriculture

Pressures:
Diffuse pollution: nutrient loading and contaminants of potential concern (e.g. pesticides);
Seasonal inflow of suspended sediments, humus and biogenic substances in mineral and organic forms (spring floods and summer storms);
Lack of control over farming operations and integrated management of Lagoon catchment area;
Water needs/supply (withdrawal of the Tyligul runoff) and consequent changes in water balance (also dependent on other conditions);
Creation of a large number of upstream artificial water bodies.

State change:
Eutrophication (seasonal and site-specific areas): Hypoxia; Periodic fish and hydrobionts kills; Reduced water transparency;
Phytoplankton and macrophyte blooms during summer;
Seasonal low water transparency, increasing water temperature in the surface layer;
Decrease floral and faunal diversity;
Changes in water balance: runoff deficit due to water management activity;
Pesticides increase in the lagoon (wash away from fields).

Positive Impacts:
Employment, wealth creation;
Viticulture and winery may be used as an additional recreational activity;
Loss of natural biodiversity (fish and other organism decline, avifauna).
Negative Impacts:
Seasonal degradation of water quality status of the lagoon (summer);
Habitat loss, natural protected areas threatened.

Responses:
Better overall regulation, law enforcement: Develop water management and monitoring plans according to the WFD.
Unique local management structure: Integrate and standardize management plans and responsible entities, inter-region regulation.
Optimization of water use: Implement programs for a sustainable use of water resources, according to local atmospheric conditions; Improve waste water treatment facilities/ plants.
Agriculture production systems and technology: Implement programmes for a sustainable development of agricutural activities (e.g. crop rotation).
RTD activities, improve knowledge to reduce uncertainty: Improve knowledge on the lagoon’s ecohydrology
Increase civic and environmental awareness: Increase public & end-users awareness; Improve local participation; Promote environmental education.

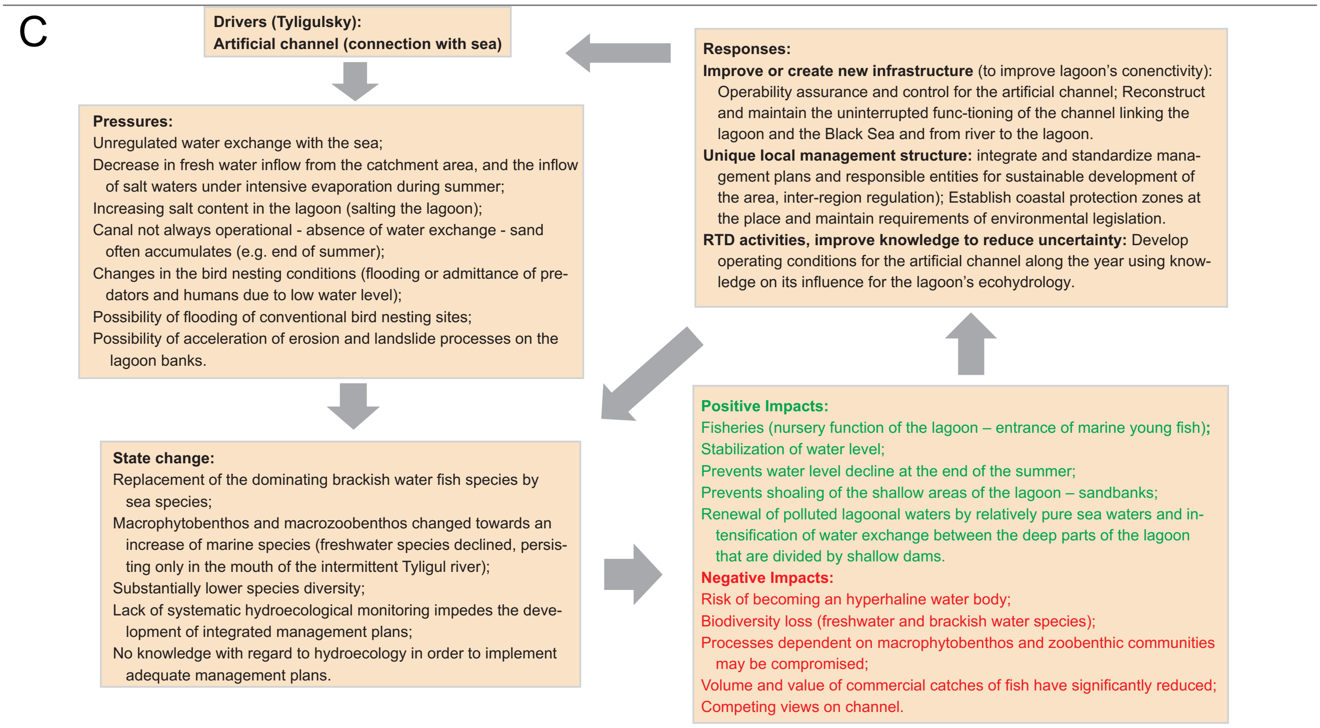

Supplementary Figure S5. DPSIR cycles for the *drivers* in the Tyligulskyi Liman Lagoon. A) Population, tourism and related activities; B) Agriculture and livestock; C) Artificial channel.

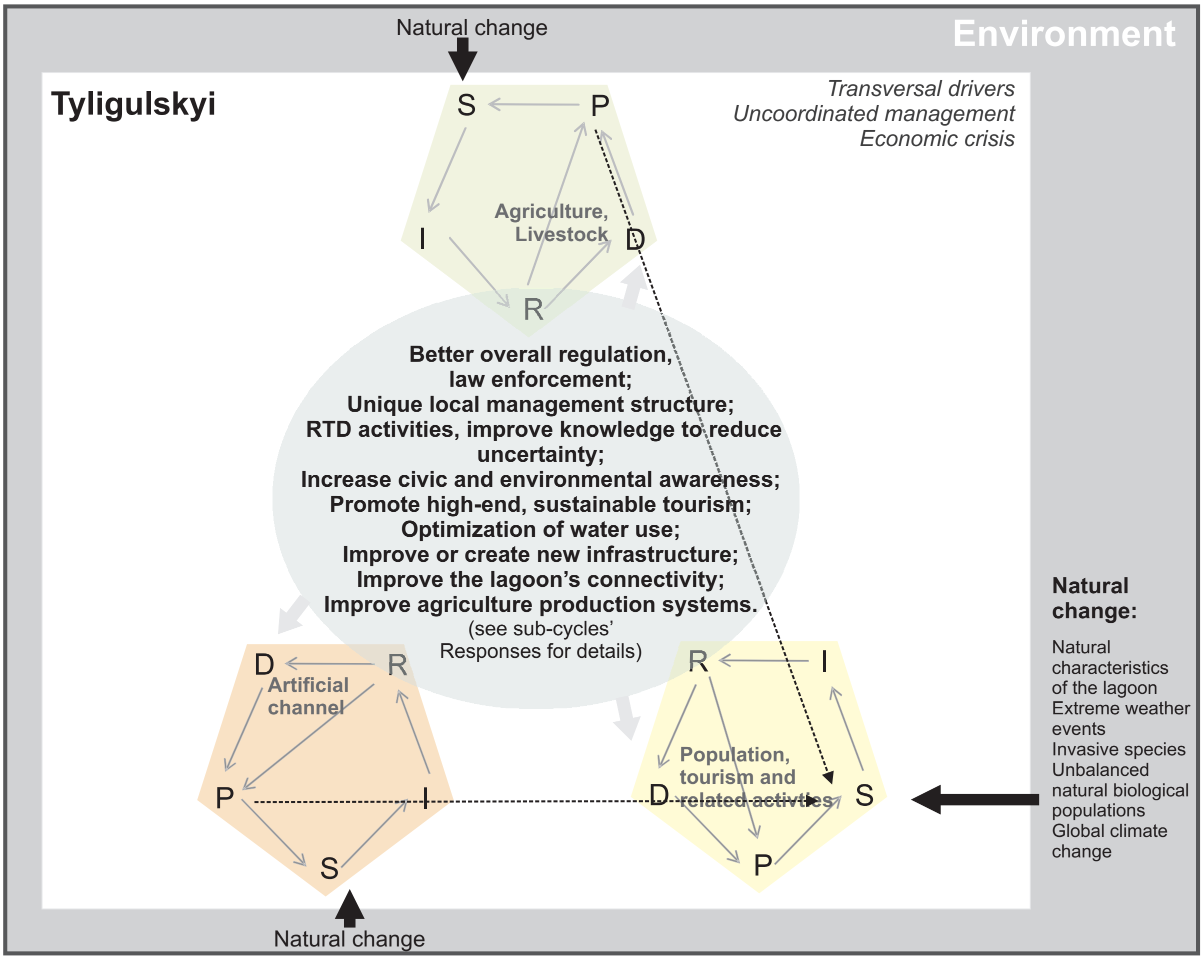

Supplementary Figure 6S. Mosaic-DPSIR for the Tylisgulskyi Lagoon, with emphasis on the *drivers* with higher social-economical expression in the lagoon, showing interactions among DPSIR cycles (dotted arrows), the influence of the natural change on the *state* (black arrows) and that *responses* from each cycle should be combined into common integrated *responses.*

DPSIR data from the Tyligulsky Lagoon available in the following references:

LAGOONS. The Tyligulski Lagoon. Current knowledge base and knowledge gaps. LAGOONS Report D2.1d. pp. (2012) Available in http://lagoons.biologiaatua.net

LAGOONS. The Tyligulskyi Lagoon, Ukraine. *LAGOONS Technical Brief TB4*. 5pp. (2012) Available in http://lagoons.biologiaatua.net

LAGOONS. Results of the problem based science analysis: the Tyligulski Lagoon, Ukraine. *LAGOONS Report D3.2.3.* (2013) Available in http://lagoons.biologiaatua.net

LAGOONS. Activities report: Report on raising public participation and awareness including design of uptake and capacity building activities. *LAGOONS Report D4.1*, 61pp. (2014). Available in http://lagoons.biologiaatua.net

Gubanova, O., *et al*. The Management Story of Tyligulskyi Liman. In Coastal Lagoons in Europe: Integrated Water Resource Strategies (Lillebø, A. I., Stalnacke, P. & Gooch, G. D.) (IWA Publishing, 2015).

Tuchkovenko Y, Loboda N, Khokhlov V. The physio-geographical background and the ecology of Tyligulskyi Liman Lagoon. In Coastal Lagoons in Europe: Integrated Water Resource Strategies (Lillebø, A. I., Stalnacke, P. & Gooch, G. D.) (IWA Publishing, 2015).

Vistula Lagoon

A

Drivers (Vistula):
Population, tourism and related activities

Pressures:
Increased population density (seasonally and locally) due to uneven touristic activity;
Waste water discharges (sometimes with insufficient treatment or not treated);
Point source pollution: contamination in the lagoon site-specific areas;
Higher touristic pressure in summer - seasonality (Polish side);
New investments into the tourism infrastructure;
Non-regulated tourism;
Incidental illegal dumping (catchment area) and mud dumping (lagoon).

State change:
Eutrophication, especially in summer: high nutrient levels, low transparency; cyanobacteria blooms;
Decrease of water and sediment quality;
Local beach disturbance (lack of maintenance, illegal mud dumping and pier construction).

Positive Impacts:
Sustainable tourism activities with economic potential to growth;
Employment and wealth creation (local economy stimulated).
Negative Impacts:
Seasonal degradation of water quality and ecological condition in the lagoon;
Potentially more pollutants in the food web, including fish.

Responses:
Better overall regulation, law enforcement: Local administration more coordinated; need for regulation of tourism activity; Need to "speed up" bureaucratic procedures related with tourism.
Promote high-end, sustainable tourism: Eco-tourism, including traditional activi-ties; Allow international transportation routes.
Improve the lagoon's navigability: Improve transport/navigation within and across the lagoon.
Increase civic and environmental awareness: environmental education and impro-ve local participation.
Optimization of water use: implementation and improvement of waste-water treatment plants.
Improve or create new infrastructure: develop coastal infrastructure to implement sustainable touristic activities and improve tourism offer (e.g. beach recreation, public transports, roads).
Restoration measures: revitalization of the sou-thern areas on the Polish side.
RTD activities, improve knowledge to reduce uncertainty: use natural resources to mitigate eutrophication (reeds, filter feeders); need for detailed assessment of the impact of the driver.

B

Drivers (Vistula)
Agriculture and livestock

Pressures:
Diffused pollution: nutrients and pesticides from agricultural and livestock sources;
Traditional value of the driver: arable land covers a substantial part of the drainage area; sector is important, but rather because of tradition and lack of alternative than the economic value it ge-nerates.

State change:
Eutrophication (trophic status of the lagoon polytrophic/eutrophic): high nutrient levels, low transparency; cyanobacteria blooms; periodic oxygen deficits at the bottom – site specific lower invertebrate diversity and seasonal decrease in density (deeper areas);
Long-term reduction of submerged vegetation;
Decreased water quality status and sediments;
Overall ecological status of Polish part (the only to be studied under WFD) classified as bad to poor.

Positive Impacts:
Employment, Wealth creation;
Social-cultural identity.
Negative Impacts:
Degradation of water quality and ecological status in the lagoon;
Impact of the lagoon degradation on the coastal waters quality of the Baltic Sea;
Negative impacts on tourism - less attractive for recreation use; Agro-tourism growth is limited due to the current water quality.

Responses:
Better overall regulation, law enforcement: Contribute to implement the WFD, CAP, Nitrate Directive, Marine Strategy (Polish side only) and respective national laws on Russian side;
RTD activities, improve knowledge to reduce uncertainty: Implement better and joint monitoring programs for the assessment of the lagoon's response to environ-mental stressors, including weather changes; Promote resilience to eutrophication impacts using lagoon's resources (e.g. reeds, filter feeder bivalves);
Transboundary harmonized management structure: better and coordinated ma-nagement, PL inter- provinces cooperation, and PL-RU transboundary cooperation;
Optimization of water use: implement and improve wastewater treatment plants.

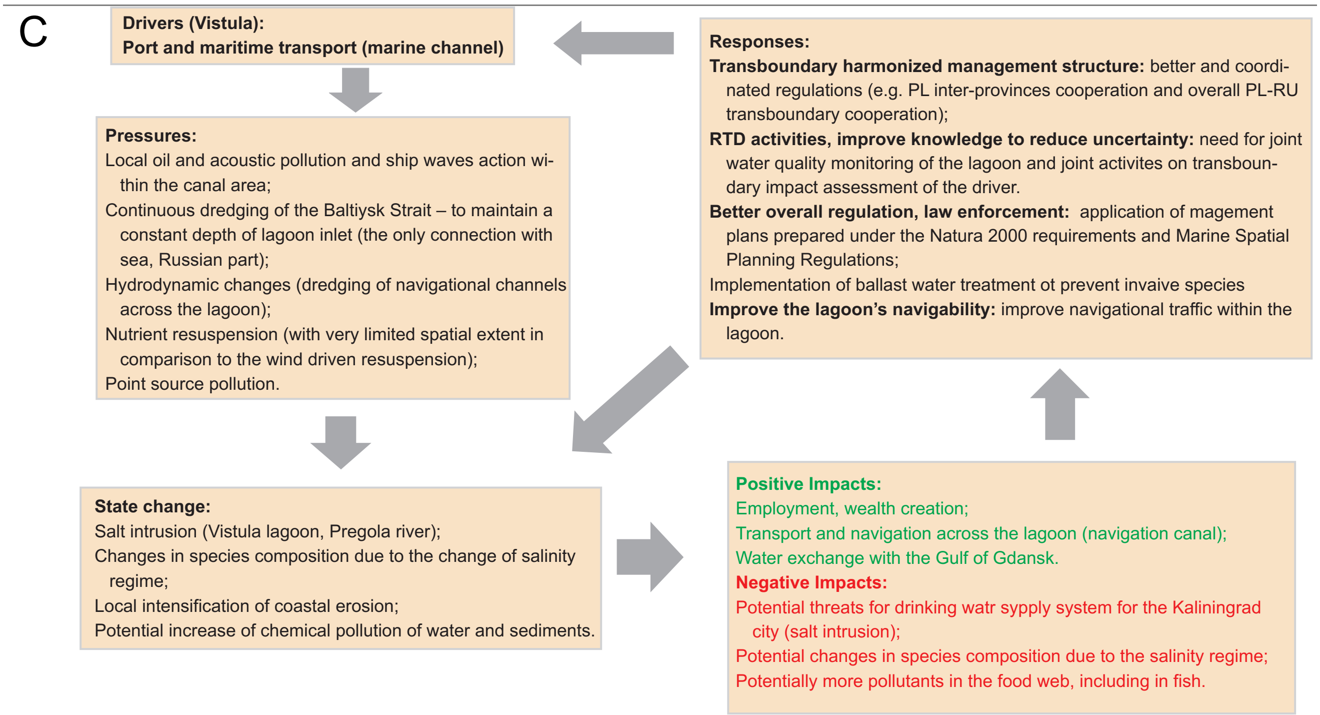

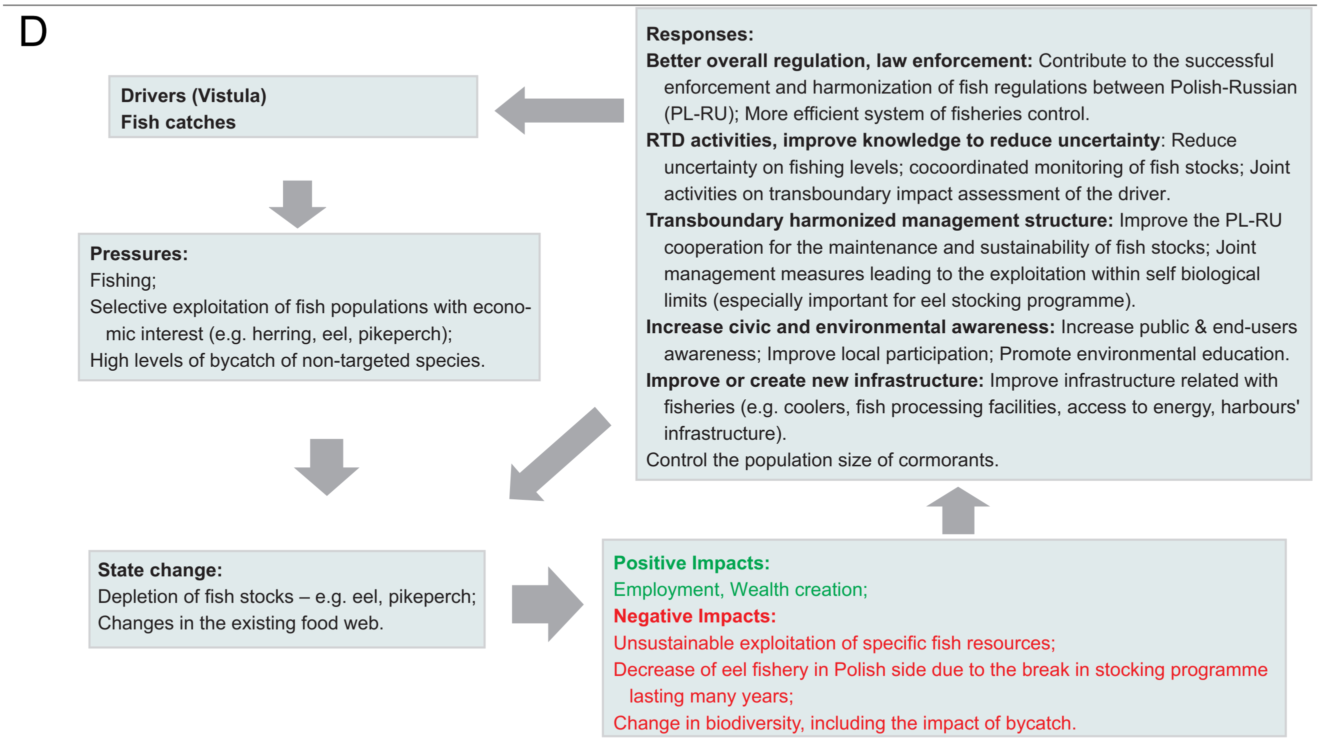

Supplementary Figure S7. DPSIR cycles for the *drivers* in the Vistula Lagoon. A) Population, tourism and related activities; B) Agriculture and livestock; C) Port and maritime transport; D) Fish catches.

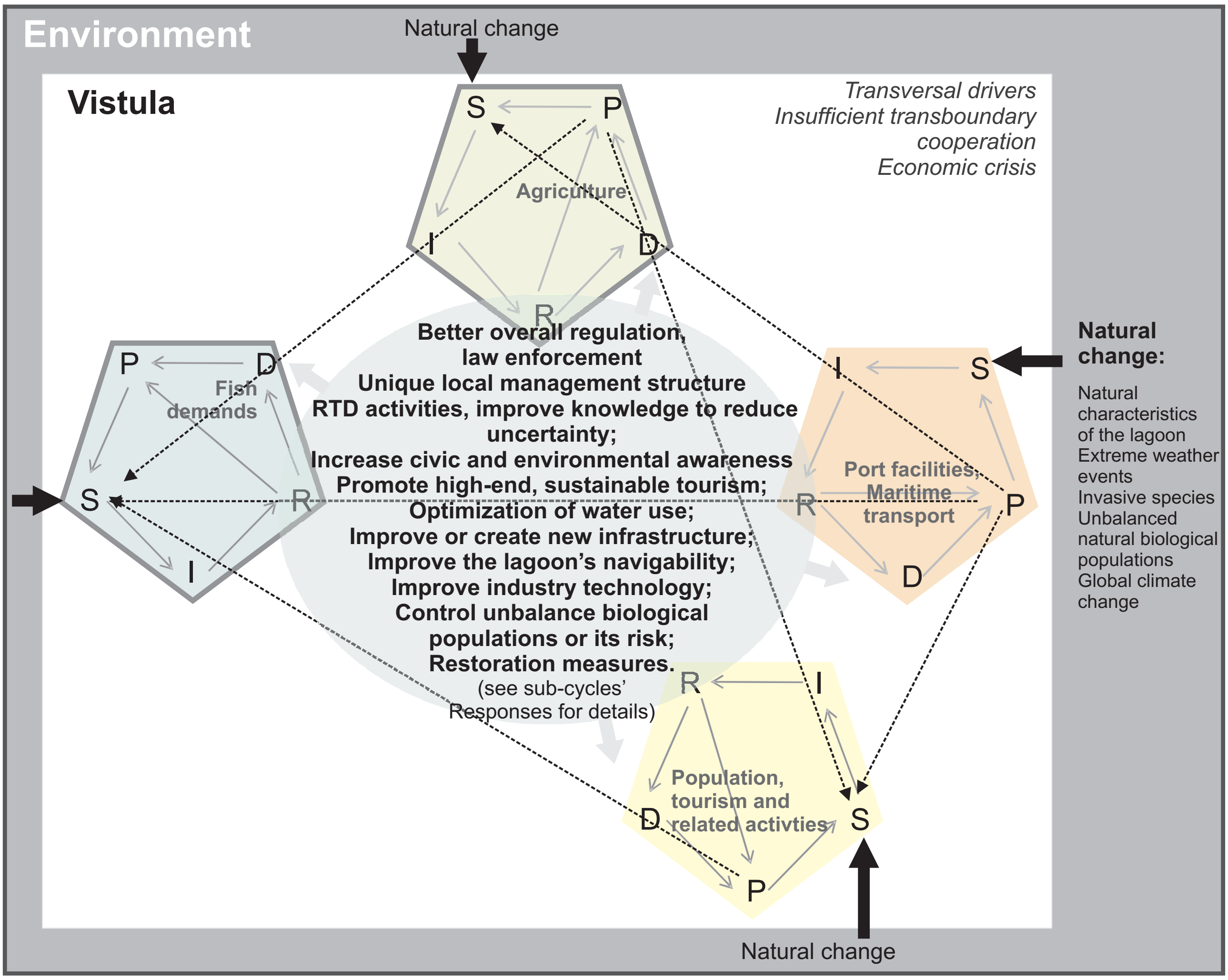

Supplementary Figure S8. Mosaic-DPSIR for the Vistula Lagoon, with emphasis on the *drivers* with higher social-economical expression in the lagoon, showing interactions among DPSIR cycles (dotted arrows), the influence of the natural change on the *state* (black arrows) and that *responses* from each cycle should be combined into common integrated *responses.*

DPSIR data from the Vistula Lagoon available in the following references:

Bielecka, M., Różyński, G. Management conflicts in the Vistula Lagoon area. *Ocean Coast Manage*, **101,** 24-34 (2014)

LAGOONS The Vistula Lagoon – Current knowledge base and knowledge gaps. LAGOONS Report D2.1a. 99pp. (2012) Available in http://lagoons.biologiaatua.net

LAGOONS The Vistula Lagoon, Poland-Russia. *LAGOONS Technical Brief* TB1. 8pp. (2012)

LAGOONS Results of the problem based science analysis: The Vistula Lagoon. *LAGOONS Report D3.2.4* 35 pp. (2013) Available in http://lagoons.biologiaatua.net

LAGOONS Grupy Fokusowe – Zalew Wiślany, Polska-Rosja. Biuletyn Proj. *LAGOONS, B4.1.,* 4pp. (2013) Available in http://lagoons.biologiaatua.net

LAGOONS Activities report: Report on raising public participation and awareness including design of uptake and capacity building activities. *LAGOONS Report D4.1*, 61pp. (2014) Available in http://lagoons.biologiaatua.net

Różyński, G., *et al*. The Physio-geographical Story of Vistula Lagoon. In Coastal Lagoons in Europe: Integrated Water Resource Strategies (Lillebø, A. I., Stalnacke, P. & Gooch, G. D.) (IWA Publishing, 2015).

Różyński, G., *et al*. The Management Story of Vistula Lagoon. In Coastal Lagoons in Europe: Integrated Water Resource Strategies (Lillebø, A. I., Stalnacke, P. & Gooch, G. D.) (IWA Publishing, 2015).
